# Synthesis and Antiproliferative Screening Of Novel Analogs of Regioselectively Demethylated Colchicine and Thiocolchicine

**DOI:** 10.3390/molecules25051180

**Published:** 2020-03-05

**Authors:** Dominika Czerwonka, Szymon Sobczak, Ewa Maj, Joanna Wietrzyk, Andrzej Katrusiak, Adam Huczyński

**Affiliations:** 1 Department of Medical Chemistry, Faculty of Chemistry, Adam Mickiewicz University, Uniwersytetu Poznańskiego 8, 61-614 Poznan, Poland; dominika.czerwonka@amu.edu.pl; 2 Department of Materials Chemistry, Faculty of Chemistry Adam Mickiewicz University, Uniwersytetu Poznańskiego 8, 61-614 Poznań, Poland; szymon.sobczak@amu.edu.pl (S.S.); katran@amu.edu.pl (A.K.); 3 Hirszfeld Institute of Immunology and Experimental Therapy, Polish Academy of Sciences, Rudolfa Weigla 12, 53-114 Wrocław, Poland; ewa.maj@hirszfeld.pl (E.M.); joanna.wietrzyk@hirszfeld.pl (J.W.)

**Keywords:** colchicine analogs, thiocolchicine, colchiceine, antimitotic agents, antiproliferative activity, hydrates

## Abstract

Colchicine, a pseudoalkaloid isolated from *Colchicum autumnale*, has been identified as a potent anticancer agent because of its strong antimitotic activity. It was shown that colchicine modifications by regioselective demethylation affected its biological properties. For demethylated colchicine analogs, 10-demethylcolchicine (colchiceine, **1**) and 1-demethylthiocolchicine (**3**), a series of 12 colchicine derivatives including 5 novel esters (**2b**–**c** and **4b**–**d**) and 4 carbonates (**2e**–**f** and **4e**–**f**) were synthesized. The antiproliferative activity assay, together with in silico evaluation of physicochemical properties, confirmed attractive biological profiles for all obtained compounds. The substitutions of H-donor and H-acceptor sites at C1 in thiocolchicine position provide an efficient control of the hydration affinity and solubility, as demonstrated for anhydrate **3**, hemihydrate **4e** and monohydrate **4a**.

## 1. Introduction

**Figure 1:**
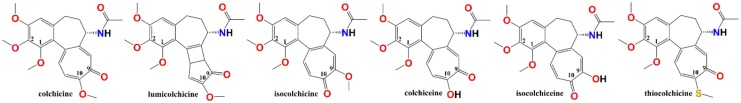
Structures of colchicine, lumicolchicine, isocolchicine, colchiceine, isocolchiceine and thiocolchicine.

Colchicine (Figure 1) is a pseudoalkaloid produced by *Colchicum autumnale* [1]. Its beneficial applications have been known for centuries, as colchicine was used by ancient Greeks and Egyptians to treat swelling and inflammation [2]. Presently, colchicine is considered as the first-line therapy for gout, pericarditis and familial Mediterranean fever [1,3,4,5]. The most interesting property of colchicine is its high affinity for binding to the tubulin in cancer cells, leading to the inhibition of microtubule polymerization and mitosis arrest [6,7,8]. These unique properties have attracted great interest to colchicine as an anticancer-drug candidate, however its use is limited by its toxicity towards normal cells [9,10,11]. For this reason, new derivatives obtained through chemical modifications guided by structure–activity relations are intensively investigated. This research is aimed at reducing the colchicine toxicity and preserving the antimitotic and anticancer properties [12,13,14,15,16,17,18,19,20,21,22,23,24]. As it turns out, tubulin interacts with trimethoxyphenyl ring A and tropolone ring C, making the methoxy groups at C1, C2 and C10, as well as the C9-keto group, crucial for colchicine’s antimitotic activity [25,26,27]. Unfortunately, colchicine is also prone to isomerization, resulting in isocolchicine (Figure 1) with 500 times lower affinity for binding to tubulin than colchicine itself [28].

Colchiceine (Figure 1) is a C10 demethylated colchicine analog characterized by much lower toxicity [29,30]. It is, next to lumicolchicine (Figure 1), the main product of colchicine degradation and can naturally occur in plant extracts together with colchicine [31]. Elguero et al. [32] have established that colchiceine (as well as its C10-acylated analog) can occur in two tautomeric forms, from which the isoform is slightly more dominant. This equilibrium between colchiceine and isocolchiceine (Figure 1) exists also in the solid state, which has been confirmed by X-ray diffraction studies [33]. The tendency of colchiceine towards tautomerization, in combination with the loss of the C10-methoxy group, may be responsible for its poor antimitotic activity. In contrast, colchiceine shows better bacteriostatic and antifungal activity than colchicine, because its free hydroxyl group may increase its binding affinity towards the cell walls of pathogens [29,33].Interestingly, the replacement of the C10-methoxy group with thiomethyl in a colchicine analog thiocolchicine (Figure 1) decreases its isomerization. Thiocolchicine is more stable, exhibits lower toxicity and has a higher binding affinity to tubulin [34,35,36]. As could be expected, demethylation of thiocolchicine at C1/C2/C3 positions leads to a lower binding ability to tubulin and lower toxicity [37,38,39]. As the removal of the methoxy groups from colchicine’s structure reduces its toxicity but also lowers its binding affinity to tubulin, we decided to replace these groups with a bigger substituent, such as an ester or carbonate moiety, to investigate an effect of this modification on biological activity [40].

**Scheme 1:**
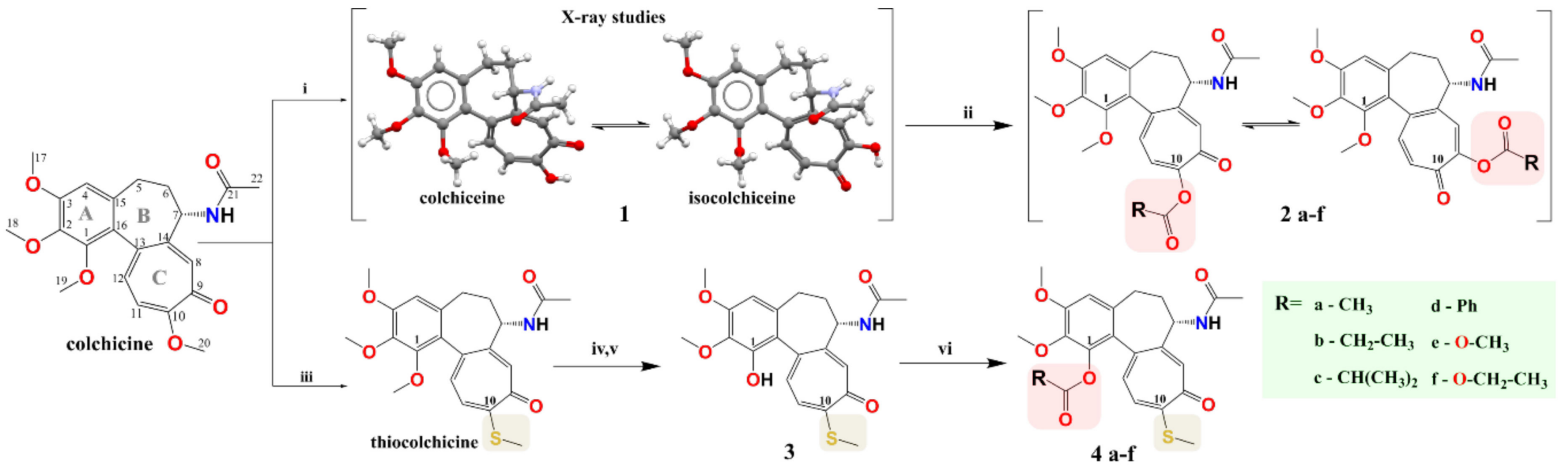
Synthesis of novel colchicine analogs. The obtained crystal structure of **1** was identical to the one proposed by Mackey et al. [33]. Reagents and conditions: (i) glacial acetic acid, hydrochloric acid, 100 °C, 6h; (iii) MeOH/H_2_O, CH_3_SNa, RT, 24h; (iv) DCM, acetyl chloride, SnCl_4_, 0 °C → RT, 48h; (v) MeOH/H_2_O, LiOH, RT, 1h; (ii,vi) DCM, Et_3_N, respective acyl chloride/chloroformate, 0 °C → RT, 24h.

For this reason, we carried out a series of regioselective demethylation reactions of colchicine and thiocolchicine, in order to to obtain colchiceine (**1**) and 1-demethylthiocolchicine (**3**). On the basis of **1** and **3**, we synthesized a series of 12 mono- and double-modified colchicine analogs. Within these analogs, we present 5 novel esters (**2b**–**c** and **4b**–**d**) and 4 novel carbonates (**2e**–**f** and **4e**–**f**). The pathways used for these syntheses are depicted in Scheme 1.

We also evaluated the antiproliferative activity of these derivatives using three human cancer cell lines, human lung adenocarcinoma (A549), human breast adenocarcinoma (MCF-7) and human colon adenocarcinoma cell line (LoVo), and one normal murine embryonic fibroblast cell line (BALB/3T3). Moreover, on the basis of the in silico calculations, we were able to predict the physicochemical properties of the obtained compounds and check their compliance with Lipiński’s rule of 5, permitting evaluation of its drug-likeness.

## 2. Results and Discussion

### 2.1. Chemistry

A series of colchicine esters and carbonates substituted at C1 and C10 positions in colchicine were synthesized by performing regioselective demethylation reactions of C1 and C10 methoxy groups, respectively. At first, we performed regioselective demethylation of the colchicine C10 methoxy group by treating colchicine with a mixture of glacial acetic acid and 0.1N hydrochloric acid (i). Reaction resulted in precipitation of yellow solid [41], which was further recrystallized in acetone, leading to growth of good quality, large single crystals. Performed X-ray diffraction analysis proved them to be a colchiceine (**1**, Scheme 1) hemihydrate of the same structure as that reported by Mackay et al. [33], in which the unit-cell accommodates two tautomeric forms: colchiceine and isocolchiceine.

**Figure 2:**
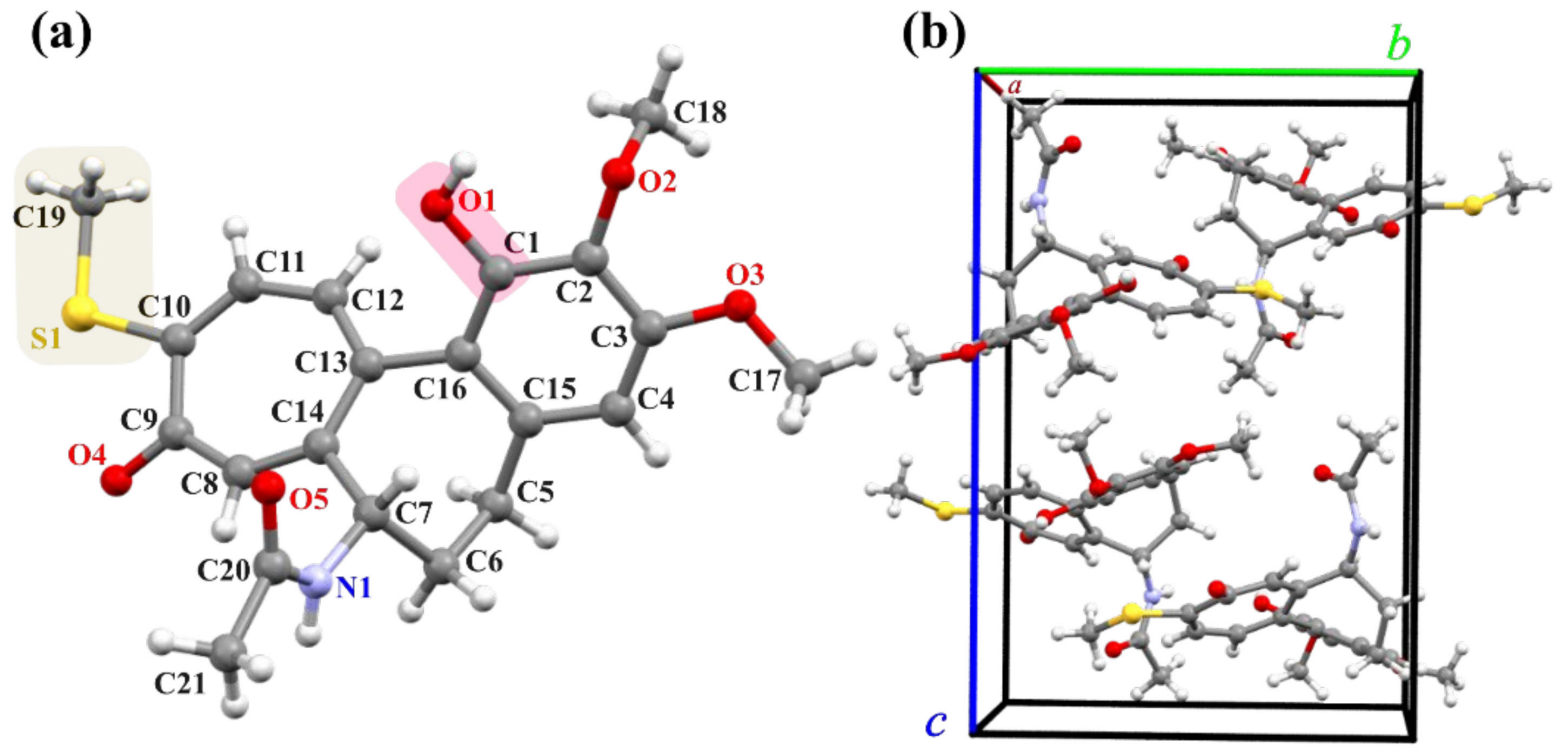
(**a**) Molecule of 1-demethylthiocolchicine (**3**) and (**b**) its crystal structure projected along the crystal direction [100].

Colchicine was also treated with sodium methanethiolate (**iii**) to obtain thiocolchicine [42]. By using a slightly modified method proposed by Bladé-Font [43] (**iv**,**v**), thiocolchicine was regioselectively demethylated at the C1 methoxy group, leading to 1-demethylthiocolchicine (**3**) with 40% yield. This regioselective demethylation was confirmed by X-ray analysis (Figure 2). Finally, compounds **1** and **3** were treated with respective acyl chlorides or chloroformates (**ii**, **vi**) to give single modified derivatives **2a**–**f** and double modified derivatives **4a**–**f** with 38%–58% yields (Scheme 1).

The purity and structure of synthesized colchicine derivatives **1**, **2a**–**f**, **3** and **4a**–**f** were determined on the basis of ESI-MS, NMR and X-ray analysis. The specific data can be found in Section 3 and in Supplementary Materials. The disappearance of the signal at about 56.5 ppm in the ^13^C NMR spectrum of colchicine, assigned to the C10 methoxy group, is the evidence of the formation of colchiceine (**1**). Moreover, in the spectra of derivatives **2a**–**2f**, an additional signal from the carbonyl group can be observed in the range 164.1–174.3 ppm for esters **2a**–**2d**, and 151.9–152.5 ppm for carbonates **2e**–**f**. A shift of the signal corresponding to the C10 methoxy group from 56.5 to 15.1 ppm is evidence for the introduction of the thiomethyl group at position C10, and the absence of one of the signals around 61 ppm assigned to the C1 methoxy group is evidence of 1-demethylthiocolchicine (**3**) formation. An additional signal corresponding to the carbonyl carbon atom can be observed in the range 168.3–174.7 ppm for esters **4a**–**4d** and in the range 152.8–152.9 ppm for carbonates **4e**–**f**.

### 2.2. In Silico Calculations of the Physicochemical Properties

We used the Molinspiration online database (http://www.molinspiration.com, free of charge) to predict the physicochemical properties of all synthesized compounds and collected them in Table 1 [44].

According to Lipinski’s rule of 5, most “drug-like” molecules should have log*P* ≤ 5, molecular weight ≤ 500, number of hydrogen bond acceptors ≤ 10 and number of hydrogen bond donors ≤ 5. Additionally, it was demonstrated that a total polar surface area (tPSA) < 140 and number of rotatable bonds < 10, in combination with log*P* < 5, are crucial for both good bioavailability and permeability through biological membranes [45].

The demethylation of the C1 and C10 position of colchicine, both in **1** and **3**, increases the number of H-donor sites while the substitution of colchicine’s C10 methoxy group with thiomethyl results in a decreased number of H-acceptor sites in **3**. Additionally, the demethylation of the C1 and C10 position of colchicine reduces the number of rotatable bonds. Substitution of a hydroxyl group, both in **1** and **3**, with an ester or carbonate substituent, increases the number of H-acceptor sites and reduces the number of H-donor sites to that of colchicine. According to the performed calculations, all synthesized compounds **2a**–**e** and **4a**–**e** have a rather lipophilic character, with the clog*P* values between 0.60 and 3.98. Conducted syntheses allowed us to obtain compounds with less lipophilic character than that of colchicine for compounds **1**, **2a** and **2e**, with clog*P* values 0.83, 060 and 0.95, respectively. Interestingly, esters and carbonates in the C10 position (**2a**–**e**) are in general less lipophilic than corresponding esters and carbonates in the C1 position (**4a**–**e**). Except for **4d**, whose molecular weight is above 500, the calculations for all colchicine analogs are in agreement with Lipinski’s descriptors. Importantly, in order to be active, a drug should not have more than one violation [46]. This, in combination with the number of rotatable bonds between 4 and 8, as well as tPSA between 84.86 and 100.18, confirm good physicochemical profiles of all obtained compounds.

### 2.3. X-ray Analysis

The structures of compounds **3**, **4a** and **4e** were additionally confirmed by X-ray diffraction. Selected crystallographic data are collected in Table 2, and more detailed information can be found in Supplementary Information. The single crystals of **3**, **4a**, **4e** were obtained by slow evaporation from an ethyl acetate mixture. The 1-Demethylthiocolchicine (**3**) crystallizes in the orthorhombic space group *P*2_1_2_1_2_1_ (Figure 2) while the crystals of **4a** and **4e** are monoclinic, space group *P*2_1_. The substitution at the C1 atom with acetyl ester (**4a**) and methyl carbonate (**4e**), and the following change in the ratio between H-bond acceptors and donors, increases the tendency of these colchicine derivatives to co-crystallize with water; hence, monohydrate **4a·**H_2_O and hemihydrate **4e·½**H_2_O were obtained.

**Figure 3:**
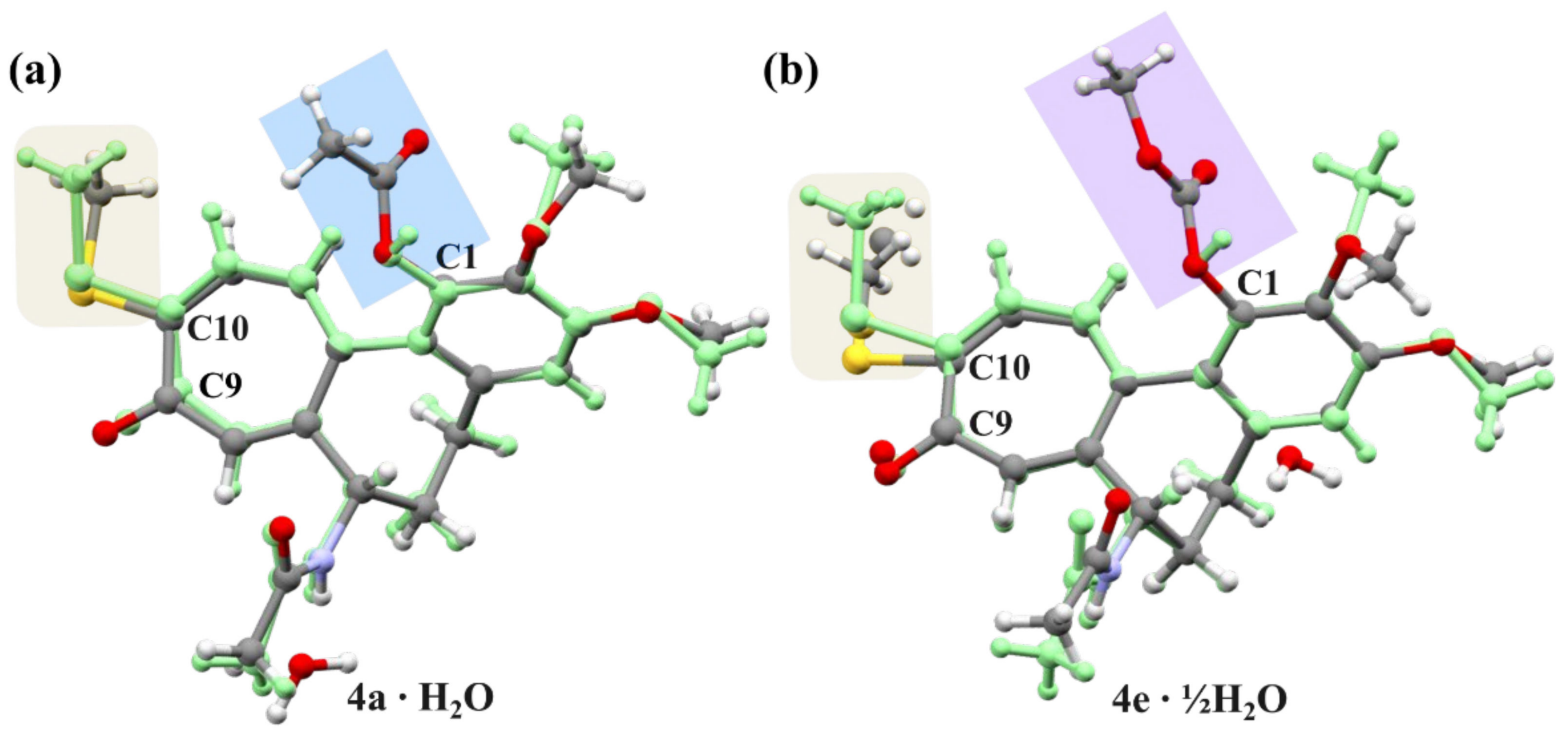
Comparison of the molecular conformation from the X-ray analysis studies between (**a**) molecule **4a** and (**b**) **4e**, superimposed with respect to the benzene ring on the 1-demethylthiocolchicine (molecule **3**), highlighted with a light-green color.

Molecules **4a** and **4e** significantly differ in conformation (Figure 3).The arrangement of the C2 methoxy group, which can be described by the C1-C2-O2-C18 torsion angle, changed from −122.36° to 108.75° in **4a** and **4e**, respectively. Together with increasing mass of the C1 moiety, the twist between the planar phenyl and tropolone rings around the C13-C16 bond increases, altering the C1-C16-C13-C12 angle from 45.43° in **3**, through 58.54° in **4a**, to 60.11° in **4e**.

The presence of water significantly alters the pattern of H-bonds between **3** and **4a·H_2_O** and **4e·½H_2_O**. Although in all structures amide nitrogen participates in the formation of intermolecular contacts, only in **3** are the NH···S, of 2.793(2) Å, and NH···O4, of 2.370(3) Å,bonds formed. In a monohydrate crystal **4a·H_2_O**, water interrupts the NH···O4 interaction observed in the crystal structure of **3**, accepting NH amide proton, of 2.086(1) Å and further donating its H-atoms to the HO-H···O4′, of 2.007(3) Å, and HO-H···O5′, of 1.994(3) Å bond. Much weaker H-bonds are present in the crystal structure of **4e·½H_2_O**. Despite the presence of a water molecule in the crystal structure of **4e·½H_2_O**, the strong NH···O5′ bond, of 2.033(2) Å is present again. The disordered by location at half-occupied sites water molecule is involved in formation of a weak HO-H···O5′’ interaction of 2.538(1) Åand the H-bond at the disordered terminal carbonyl at C9. Due to the HO-H···O4′ of 2.241(1) Å bond, the occupational disorder of H_2_O is passed onto the orientation of **4e** molecules as illustrated in Figure 3. Depending on the presence or absence of this water molecule, its adjacent molecule **4e** is slightly shifted in the crystal structure. This disorder is most apparent for the terminal carbonyl at C9 (dislocated by 0.358 Å) and thiomethyl at C10 (dislocated by 0.625 Å).

### 2.4. Antiproliferative Activity

The antiproliferative activity of all synthesized colchicine analogs **1**, **2a**–**f**, **3**, **4a**–**f** was *tested in vitro* against three human cancer cell lines: human lung adenocarcinoma (A549), human breast adenocarcinoma (MCF-7) and human colon adenocarcinoma cell line (LoVo). For a more accurate evaluation of cytotoxic activity, the effect on normal murine embryonic fibroblast cell line (BALB/3T3) was also tested according to the previously published procedure [12]. Detailed information concerning antiproliferation assay can be found in Supplementary Materials. The mean values of IC_50_ ± SD of the tested compounds are collected in Table 3.

All colchicine analogs showed stronger antiproliferative activity against all tested cancer cell lines than the conventional chemotherapeutic, cisplatin. In general, the IC_50_ values are better for colchicines doubly modified at C1 and C10 positions (**3**, **4a**–**f**) than for the analogs singly modified at position C10 only (**1**, **2a**–**f**). Compound **4a**, with a thiomethyl group at position C10 and an acetyl ester substituent at C1, showed the highest activity against all tested cell lines (IC_50_ = 0.48 ± 0.15, 0.10 ± 0.02, 0.11 ± 0.02 μM for A549, MCF-7, LoVo cancer cell lines, respectively).

All tested colchicine analogs exhibit higher SI values than doxorubicin, which indicates the therapeutic potential of the synthesized compounds. Compound **3**, 1-demethylthiocolchicine, as well as its acylated analog **4a**, showed high SI values against MCF-7 (SI = 4.92, SI = 6.88) as well as LoVo cell lines (SI = 5.36, SI = 5.95). From among analogs modified exclusively at position C10, compound **2b** with propionyl ester substituent revealed a higher SI value towards LoVo cell line (SI = 3.13).

## 3. Materials and Methods

### 3.1. General

Information concerning reagents and solvents, as well as equipment used for measurements, can be found in the Supplementary Materials.

#### 3.1.1. Synthesis and Characterization of 10-Demethylcolchicine (colchiceine, 1), Thiocolchicine and 1-Demethylthiocolchicine (3)

Information concerning the synthesis of 10-demethylcolchicine (colchiceine, **1**), thiocolchicine and 1-demethylthiocolchicine (**3**) can be found in Supplementary Materials.

#### 3.1.2. General Route for Synthesis of Compounds 2a–f and 4a–f

To a solution of **1** or **3** (100 mg, 0.25 mmol) in dichloromethane (DCM, 10 mL) cooled to 0 °C, Et_3_N (1 mL, 7 mmol) and respective acyl chloride or chloroformate were added (excess). The mixture was first stirred at 0 °C for 30 min and then for the next 24h at RT. Reaction time was determined by TLC. After that time the solution was filtered to remove triethylamine hydrochloride, the DCM was evaporated under reduced pressure and the residue was purified by CombiFlash^®^ (chloroform/acetone, increasing concentration gradient) to give respective compounds as amorphous yellow solids.

#### 3.1.3. Characterization of Acetyl Ester of Colchiceine 2a

Amorphous yellow solid, yield 49%, m.p 120–122 °C; ^1^H NMR (CDCl_3_, 400 MHz) δ 7.50 (1H, s), 7.31 (1H, d, *J* = 11.3 Hz), 7.22 (1H, d, *J* = 11.3 Hz), 6.53 (1H, s), 4.60 (1H, dt, *J* = 12.7, 6.5 Hz), 3.91 (3H, s), 3.88 (3H, s), 3.66 (3H, s, 2.50 (1H, dd, J = 13.6, 6.3 Hz), 2.35 (3H, s), 2.26–2.17 (1H, m), 1.96 (3H, s), 1.87 (1H, td, *J* = 12.1, 6.9 Hz). ^13^C NMR (CDCl_3_, 101 MHz) δ 169.9, 168.2, 153.9, 151.0, 148.6, 141.8, 141.5, 137.0, 134.5, 131.2, 130.3, 125.2, 107.4, 61.6, 61.3, 56.0, 52.1, 36.8, 29.7, 22.7, 20.7. ESI-MS (m/z): [M + H]^+^ 428, [M + Na]^+^ 450, [M + K]^+^ 466, [2M + Na]^+^ 877.

#### 3.1.4. Characterization of Propionyl Ester of Colchiceine 2b

Amorphous yellow solid, yield 52%, m.p 133-135 °C; ^1^H NMR (CDCl_3_, 400 MHz) δ 7.50 (1H, s), 7.30 (1H, d, *J* = 11.3 Hz), 7.22 (1H, d, *J* = 11.2 Hz), 6.52 (1H, s), 4.66–4.57 (1H, m), 3.91 (3H, s), 3.88 (3H, s), 3.66 (3H, s), 2.66 (2H, q, *J* = 7.4 Hz), 2.50 (1H, dd, *J* = 13.5, 6.3 Hz), 2.35 (1H, ddd, *J* = 12.7, 10.3, 6.0 Hz), 2.24–2.16 (1H, m), 1.97 (3H, s), 1.87 (2H, td, *J* = 12.0, 6.7 Hz), 1.26 (3H, t, *J* = 7.6 Hz). ^13^C NMR (CDCl_3_, 101 MHz) δ 171.8, 169.9, 153.9, 151.1, 148.9, 141.7, 141.6, 136.7, 134.5, 130.9, 130.5, 125.2, 107.4, 61.7, 61.4, 56.1, 52.1, 36.9, 29.8, 27.4, 22.8, 9.0. ESI-MS (m/z): [M + Na]^+^ 464, [2M + Na]^+^ 905.

#### 3.1.5. Characterization of Isobutyryl Ester of Colchiceine 2c

Yellowish brown oil, yield 55%; ^1^H NMR (CDCl_3_, 400 MHz) δ 7.56 (1H, s), 7.28 (1H, d, *J* = 11.0 Hz), 7.23 (1H, d, *J* = 10.6 Hz), 6.52 (1H, s), 4.64 (1H, dt, *J* = 12.2, 6.8 Hz), 3.91 (3H, s), 3.88 (3H, s), 3.65 (3H, s), 2.90–2.82 (1H, m), 2.54–2.47 (1H, m), 2.36 (1H, td, *J* = 13.2, 6.9 Hz), 2.24–2.12 (2H, m), 1.96 (3H, s), 1.92–1.85 (1H, m), 1.35 (3H, d, *J* = 1.4 Hz), 1.33 (3H, d, *J* = 1.3 Hz). ^13^C NMR (CDCl_3_, 101 MHz) δ 174.3, 170.0, 153.9, 151.1, 150.3, 142.1, 141.5, 136.0, 134.5, 131.6, 130.1, 125.2, 107.4, 61.7, 61.3, 56.0, 52.1, 36.6, 34.0, 29.8, 26.4, 22.7, 19.0. ESI-MS (m/z): [M + Na]^+^ 478, [2M + Na]^+^ 933.

#### 3.1.6. Characterization of Benzyl Ester of Colchiceine 2d

Amorphous yellow solid, yield 41%, m.p 123–125 °C; ^1^H NMR (CDCl_3_, 400 MHz) δ 8.18 (2H, d, *J* = 7.4 Hz), 7.65–7.60 (2H, m), 7.49 (1H, ddd, *J* = 4.8, 3.1, 1.1 Hz), 7.34 (2H, s), 6.53 (1H, s), 4.66–4.58 (1H, m), 3.93 (3H, d, *J* = 0.9 Hz,), 3.90 (3H, d, *J* = 0.7 Hz), 3.68 (3H, d, *J* = 1.1 Hz), 2.49 (1H, dd, *J* = 13.4, 4.9 Hz), 2.42–2.32 (1H, m), 2.17–2.06 (1H, m), 1.86 (3H, d, *J* = 4.0 Hz), 1.84–1.78 (1H, m). ^13^C NMR (CDCl_3_, 101 MHz) δ 169.9, 164.1, 153.9, 151.1, 142.0, 141.6, 134.5, 133.8, 130.4, 128.9, 128.5, 125.3, 107.4, 61.7, 61.4, 56.1, 52.0, 36.9, 29.8, 22.7. ESI-MS (m/z): [M + H]^+^ 490, [M + Na]^+^ 512, [M + K]^+^ 528.

#### 3.1.7. Characterization of Methyl Carbonate of Colchiceine 2e

Amorphous yellowish brown solid, yield 58%, m.p 115–118 °C; ^1^H NMR (CDCl_3_, 400 MHz) δ 7.57 (1H, s), 7.32 (1H, d, *J* = 20.5 Hz), 6.54 (1H, s), 4.62 (1H, dt, *J* = 11.8, 6.0 Hz), 3.92 (3H, s)*, 3.92 (3H, s)*, 3.89 (3H, s), 3.68 (3H, s), 2.52 (1H, dd, *J* = 13.4, 6.1 Hz), 2.37 (1H, td, *J* = 13.0, 6.7 Hz), 2.29–2.18 (1H, m), 1.99 (3H, s), 1.91 (1H, dd, *J* = 19.0, 12.2 Hz). ^13^C NMR (CDCl_3_, 101 MHz) δ 169.9, 154.1, 152.5, 151.1, 141.6, 134.5, 125.1, 107.4, 61.7, 61.3, 56.1, 55.8, 52.2, 37.2, 29.8, 22.8. ESI-MS (m/z): [M + H]^+^ 444, [M + Na]^+^ 466, [M + K]^+^ 482, [2M + Na]^+^ 909.

#### 3.1.8. Characterization of Ethyl Carbonate of Colchiceine 2f

Yellowish brown oil, yield 55%; ^1^H NMR (CDCl_3_, 400 MHz) *δ* 7.46 (1H, s), 7.40–7.32 (1H, m, *J* = 6.2 Hz), 7.30–7.21 (1H, m), 6.55 (1H, s), 4.62 (1H, dt, *J* = 12.6, 6.4 Hz), 4.36 (2H, q, *J* = 7.1 Hz), 3.93 (3H, s, *J* = 2.9 Hz), 3.90 (3H, s), 3.68 (3H, s), 2.54 (1H, dd, *J* = 13.6, 6.4 Hz), 2.41 (1H, td, *J* = 13.1, 7.1 Hz), 2.32–2.21 (1H, m), 2.01 (3H, s), 1.94–1.84 (1H, m), 1.41 (3H, t, *J* = 7.1 Hz). ^13^C NMR (CDCl_3_, 101 MHz) *δ* 169.9, 154.0, 151.9, 151.1, 141.6, 134.5, 125.1, 107.4, 65.4, 61.7, 61.3, 56.1, 52.1, 37.2, 29.8, 22.8, 14.1. ESI-MS (*m/z*): [M + H]^+^ 458, [M + Na]^+^ 480, [M + K]^+^ 496, [2M + Na]^+^ 937.

#### 3.1.9. Characterization of Acetyl Ester of 1-demethylthiocolchicine 4a

Amorphous yellow solid, yield 45%, m.p 168-172 °C; ^1^H NMR (DMSO-d_6_, 400 MHz) δ 8.51 (1H, d, *J* = 7.7 Hz), 7.23 (1H, d, *J* = 10.8 Hz), 7.02 (1H, s), 6.97 (1H, s), 6.92 (1H, d, *J* = 10.3 Hz), 3.89 (3H, s), 3.74 (3H, s), 2.65 (1H, dd, *J* = 13.5, 6.0 Hz), 2.41 (3H, s), 2.32–2.24 (1H, m), 2.22 (3H, s), 2.12–2.01 (1H, m), 1.85 (3H, s, *J* = 6.3 Hz), 1.84–1.77 (1H, m, *J* = 12.1, 6.8 Hz). ^13^C NMR (DMSO-d_6_, 101 MHz) δ 181.2, 168.9, 168.2, 157.7, 152.8, 150.4, 140.8, 139.3, 136.4, 134.4, 132.7, 128.2, 126.4, 124.6, 109.9, 60.1, 56.0, 50.7, 36.2, 29.1, 22.5, 20.3, 14. ESI-MS (m/z): [M + Na]^+^ 466, [2M + Na]^+^ 909.

#### 3.1.10. Characterization of Propionyl Ester of 1-demethylthiocolchicine 4b

Amorphous yellow solid, yield 47%, m.p 136-140 °C; ^1^H NMR (DMSO-d_6_, 400 MHz) δ 8.52 (1H, d, *J* = 7.7 Hz), 7.23 (1H, d, *J* = 10.7 Hz), 7.03 (1H, s), 6.98 (1H, s), 6.92 (1H, d, *J* = 10.2 Hz), 4.34 (1H, dt, *J* = 11.8, 7.1 Hz), 3.90 (3H, s), 3.76–3.71 (3H, m), 2.66 (1H, dd, *J* = 13.2, 6.4 Hz), 2.41 (3H, s), 2.33–2.19 (2H, m), 2.08 (2H, ddd, *J* = 11.4, 9.8, 5.4 Hz), 1.84–1.78 (1H, m), 1.11 (3H, t, *J* = 7.5 Hz). ^13^C NMR (DMSO-d_6_, 101 MHz) δ 181.2, 172.3, 168.3, 157.7, 152.8, 150.5, 140.9, 139.2, 136.4, 134.4, 132.9, 128.3, 126.4, 124.7, 109.9, 60.1, 56.0, 50.7, 36.2, 29.1, 26.7, 22.6, 14.5, 9.2. ESI-MS (m/z): [M + H]^+^ 458, [M + Na]^+^ 480, [M + K]^+^ 496, [2M + Na]^+^ 937.

#### 3.1.11. Characterization of Isobutyryl Ester of 1-demethylthiocolchicine 4c

Amorphous yellow solid, yield 49%, m.p 165-167 °C; ^1^H NMR (DMSO-d_6_, 101 MHz) δ 8.50 (1H, d, *J* = 7.8 Hz), 7.22 (1H, d, *J* = 10.6 Hz), 7.03 (1H, s), 6.98 (1H, s), 6.91 (1H, d, *J* = 8.8 Hz), 4.35 (1H, dt, *J* = 11.7, 7.3 Hz), 3.90 (3H, s), 3.72 (3H, s), 2.74 (1H, dt, *J* = 13.9, 6.9 Hz), 2.66 (1H, dd, *J* = 13.6, 6.1 Hz), 2.39 (3H, s), 2.34–2.25 (1H, m), 2.09 (1H, td, *J* = 12.7, 5.9 Hz), 1.86 (3H, s), 1.81 (1H, dd, *J* = 12.0, 6.9 Hz, 1.16 (3H, d, *J* = 3.5 Hz), 1.14 (3H, d, *J* = 3.5 Hz). ^13^C NMR (DMSO-d_6_, 101 MHz) δ 181.2, 174.7, 168.3, 157.7, 152.7, 150.4, 140.8, 139.2, 136.4, 134.4, 132.9, 128.3, 126.3, 124.8, 109.9, 60.1, 56.1, 50.7, 36.2, 33.3, 29.1, 22.6, 18.8, 14.6. ESI-MS (m/z): [M + Na]^+^ 494, [2M + Na]^+^ 965.

#### 3.1.12. Characterization of Benzyl Ester of 1-demethylthiocolchicine 4d

Amorphous yellow solid, yield 38%, m.p 166-170 °C; ^1^H NMR (DMSO-d_6_, 101 MHz) δ 8.52 (1H, s), 8.07 (2H, d, *J* = 7.3 Hz), 7.77–7.71 (1H, m), 7.63–7.56 (2H, m), 7.22–7.12 (1H, m), 7.06 (1H, s), 7.02 (1H, s), 4.49–4.40 (1H, m), 3.94 (3H, s), 3.74 (3H, s), 2.72 (1H, dd, *J* = 13.0, 5.9 Hz), 2.36 (1H, dd, *J* = 7.4, 5.5 Hz,), 2.31 (3H, s), 2.13 (2H, ddd, *J* = 19.4, 12.8, 6.9 Hz,), 1.87 (3H, s).^13^C NMR (DMSO-d_6_, 101 MHz) δ 181.1, 168.3, 157.7, 152.9, 150.6, 141.0, 139.4, 136.3, 134.6, 134.2, 129.8, 129.1, 128.4, 128.3, 126.2, 124.9, 110.2, 60.3, 56.1, 50.8, 36.3, 29.2, 22.6, 14.5. ESI-MS (m/z): [M + Na]^+^ 528, [2M + Na]^+^ 1033.

#### 3.1.13. Characterization of Methyl Carbonate of 1-demethylthiocolchicine 4e

Amorphous yellow solid, yield 53%, m.p 158-162 °C; ^1^H NMR (DMSO-d_6_, 101 MHz) δ 8.55 (1H d, *J* = 7.6 Hz), 7.26 (1H, d, *J* = 10.6 Hz), 7.05–6.99 (3H, m), 4.34 (1H, dt, *J* = 11.8, 7.0 Hz), 3.91 (3H, s), 3.82 (3H, s), 3.79 (3H, s), 2.69 (1H, dd, *J* = 13.5, 6.2 Hz), 2.43 (3H, s), 2.29 (1H, ddd, *J* = 18.6, 12.3, 5.4 Hz), 2.09 (1H, ddd, *J* = 18.7, 12.5, 6.2 Hz), 1.88 (3H, s), 1.86–1.80 (1H, m). ^13^C NMR (DMSO-d_6_, 101 MHz) δ 181.2, 168.4, 157.9, 152.9, 152.8, 150.5, 140.8, 139.3, 136.0, 134.5, 133.2, 128.2, 126.4, 124.3, 110.2, 60.4, 56.14, 56.07, 50.8, 36.1, 29.1, 22.6, 14.4. ESI-MS (m/z): [M + H]^+^ 460, [M + Na]^+^ 482, [M + K]^+^ 498, [2M + Na]^+^ 941.

#### 3.1.14. Characterization of Ethyl Carbonate of 1-demethylthiocolchicine 4f

Amorphous yellow solid, yield 48%, m.p 165-167 °C; ^1^H NMR (DMSO-d_6_, 400 MHz) δ 8.52 (1H, d, *J* = 7.6 Hz), 7.23 (1H, d, *J* = 10.7 Hz), 7.01 (3H, t, *J* = 10.3 Hz), 4.33 (1H, dt, *J* = 12.6, 7.5 Hz), 4.27–4.20 (2H, m), 3.90 (3H, s), 3.78 (3H, s), 2.67 (1H, dd, *J* = 13.2, 6.6 Hz), 2.41 (3H, s), 2.34–2.23 (1H, m), 2.08 (3H, s), 1.87 (3H, s), 1.85–1.78 (1H, m), 1.22 (3H, t, *J* = 7.1 Hz). ^13^C NMR (DMSO-d_6_, 101 MHz) δ 181.2, 168.4, 157.9, 152.8, 152.4, 150.5, 140.8, 139.3, 136.2, 134.5, 133.1, 128.2, 126.3, 124.4, 110.2, 65.1, 60.3, 56.1, 50.8, 36.1, 29.1, 22.6, 14.4, 14.0. ESI-MS (m/z): [M + Na]^+^ 496, [M + K]^+^ 512, [2M + Na]^+^ 969.

### 3.2. Antiproliferative Activity

Detailed information concerning antiproliferative activity assay is given in Supplementary Materials.

### 3.3. X-ray Measurements

X-ray measurements and detailed crystallographic data are given in Supplementary Materials.

## 4. Conclusions

On the basis of regioselectively demethylated colchicine analogs, colchiceine (**1**) and 1-demethylthiocolchicine (**3**), we have designed and synthesized a series of **12** colchicine derivatives bearing ester and carbonate substituents, including **9** entirely novel derivatives (**2b**,**c**,**e**,**f** and **4b**,**c**,**d**,**e**,**f**). Derivatives **4a**·**H_2_O** and **4e**·**½H_2_O**, with acetyl ester and methyl carbonate moieties, display reduced hydrophilic properties and crystallize as hydrates, which affects their biological activity and increases their solubility. The control over water co-crystallization from monohydrate to hemihydrate has been achieved through the number of H-donor and acceptor sites. These features can be invaluable for the regulation of the bioaccessibility and pharmaceutical processing of this group of compounds [47]. The synthesized derivatives exhibit a considerable in vitro antiproliferative activity against three human cancer cell lines. Compound 4a, carrying the thiomethyl group at position C10 and acetyl ester substituent at C1, showed the highest activity and selectivity index values. The biological evaluation has been supported by prediction of physicochemical properties, which are consistent with Lipinski’s rule of five for all synthesized analogs.

## Figures and Tables

**Table 1:** Physicochemical properties of the synthesized compounds based on the Molinspiration database [44].

Compound	MW	clog*P*	tPSA	n(O,N)	N(OH,NH)	Rotb	MV
**1**	385.42	0.83	94.10	7	2	4	346.63
**2a**	427.45	0.60	100.18	8	1	6	383.14
**2b**	441.48	1.27	100.18	8	1	7	399.94
**2c**	455.51	1.51	100.18	8	1	7	416.53
**2d**	489.52	2.92	100.18	8	1	7	437.99
**2e**	443.45	0.95	109.41	9	1	7	392.12
**2f**	457.48	1.33	109.41	9	1	8	408.93
**3**	401.48	1.89	84.86	6	2	4	355.77
**4a**	443.52	1.66	90.94	7	1	6	392.28
**4b**	457.55	2.33	90.94	7	1	7	409.08
**4c**	471.57	2.57	90.94	7	1	7	425.67
**4d**	505.59	3.98	90.94	7	1	7	447.13
**4e**	459.52	2.02	100.18	8	1	7	401.27
**4f**	473.55	2.39	100.18	8	1	8	418.07
**colchicine**	399.44	1.10	83.11	7	1	5	364.15

MW: Molecular weight; clog*P*: calculated log octanol/water partition coefficient; tPSA: total polar surface area; n(O,N): number of hydrogen acceptors; n(OH,NH): number of hydrogen donors; Rotb: rotatable bonds; MV: molecular volume.

**Table 2:** Selected crystallographic data for compounds **3**, **4a·H_2_O** and **4e·½H_2_O**.

Label		3	4a·H_2_O	4e·½H_2_O
**Formula**		C_21_H_22_NO_5_S	C_23_H_25_NO_6_S · H_2_O	C_23_H_25_NO_7_S· ½H_2_O
**CCDC number**		1966196	1966194	1966195
Crystal system		orthorhombic	monoclinic	monoclinic
Space group		*P*2_1_2_1_2_1_	*P*2_1_	*P*2_1_
Unit cell dimensions	*a* (Å)	9.1005(17)	10.600(2)	10.8835(10)
	*b* (Å)	11.866(2)	6.9635(11)	9.2158(6)
	*c* (Å)	17.881(4)	16.712(3)	12.0632(12)
	*β* (°)	90	107.07(2)	104.613(9)
Volume (Å^3^)		1930.8(7)	1179.2(4)	1170.80(18)
*Z/Z’*		4/1	2/1	2/1
*D*_x_ (g/cm^3^)		1.378	1.300	1.329

**Table 3:** Antiproliferative activity (IC_50_) of 10-demethylcolchicine (colchiceine, **1**), 1-demethylthiocolchicine (**3**) and their derivatives(**2a**–**f**, **4a**–**f**) compared to the antiproliferative activity of colchicine and standard anticancer drugs doxorubicin and cisplatin and the calculated values of selectivity index (SI).

Compound	A549	SI	MCF-7	SI	LoVo	SI	BALB/3T3
	IC_50_ (μM)		IC_50_ (μM)		IC_50_ (μM)		IC_50_ (μM)
**1**	12.99 ± 1.79	0.79	11.23 ± 2.52	0.91	6.00 ± 1.88	1.71	10.25 ± 0.96
**2a**	12.47 ± 2.77	0.68	10.03 ± 2.18	0.85	6.18 ± 1.48	1.37	8.50 ± 0.50
**2b**	9.97 ± 0.70	0.75	7.56 ± 2.84	0.99	2.39 ± 1.20	3.13	7.47 ± 0.50
**2c**	20.39 ± 11.30	0.41	11.95 ± 1.54	0.70	6.47 ± 0.35	1.30	8.42 ± 1.14
**2d**	8.75 ± 0.33	1.02	8.21 ± 3.35	1.09	4.87 ± 2.17	1.83	8.92 ± 3.93
**2e**	0.98 ± 0.16	0.84	0.98 ± 0.33	0.84	0.48 ± 0.32	1.70	0.82 ± 0.11
**2f**	9.67 ± 0.84	0.86	9.07 ± 1.90	0.92	6.41 ± 1.57	1.30	8.36 ± 0.17
**3**	0.82 ± 0.02	0.74	0.12 ± 0.05	4.92	0.11 ± 0.03	5.38	0.61 ± 0.14
**4a**	0.48 ± 0.15	1.36	0.10 ± 0.02	6.85	0.11 ± 0.02	5.92	0.65 ± 0.12
**4b**	0.97 ± 0.04	0.77	0.96 ± 0.13	0.78	0.50 ± 0.19	1.53	0.75 ± 0.10
**4c**	0.89 ± 0.07	0.80	0.86 ± 0.10	0.83	0.56 ± 0.06	1.27	0.71 ± 0.03
**4d**	0.60 ± 0.31	0.75	0.78 ± 0.22	0.58	0.10 ± 0.08	4.71	0.45 ± 0.23
**4e**	0.90 ± 0.09	0.87	1.02 ± 0.10	0.77	0.43 ± 0.19	1.84	0.78 ± 0.06
**4f**	0.96 ± 0.13	1.08	0.95 ± 0.20	1.09	0.79 ± 0.16	1.31	1.03 ± 0.20
**colchicine**	0.07 ± 0.01	0.63	0.01 ± 0.01	4.30	0.01 ± 0.01	5.38	0.04 ± 0.01
**cisplatin**	3.60 ± 0.25	0.81	3.05 ± 0.67	0.95	3.80 ± 0.28	0.77	2.91 ± 1.83
**doxorubicin**	0.16 ± 0.03	0.25	0.15 ± 0.05	0.26	0.08 ± 0.03	0.49	0.04 ± 0.03

The IC_50_ value is defined as the concentration of a compound at which 50% growth inhibition is observed. Human lung adenocarcinoma (A549), human breast adenocarcinoma (MCF-7), human colon adenocarcinoma cell line (LoVo) and normal murine embryonic fibroblast cell line (BALB/3T3). The SI (Selectivity Index) was calculated for each compound, using the formula SI = IC_50_ for normal cell line BALB/3T3/IC_50_ for the respective cancerous cell line. A beneficial SI > 1.0 indicates a drug with efficacy against tumor cells greater than the toxicity against normal cells.

## Supplementary Materials

The following are available online: General procedures, experimental details, as well as an elaborate description of the X-ray diffraction sudies and in vitro methods used.
