# Supplementary material for: Synthesis and Antiproliferative Screening Of Novel Analogs of Regioselectively Demethylated Colchicine and Thiocolchicine
Source: Molecules. 2020 Mar 5;25(5):1180. doi: 10.3390/molecules25051180 (PMC7179419; doi:10.3390/molecules25051180)
Supplement: Supplementary file 1 [file molecules-25-01180-s001.pdf]

Synthesis and antiproliferative screening of novel analogs of regioselectively demethylated colchicine and thiocolchicne

Dominika Czerwonka [a], Szymon Sobczak [b], Ewa Maj [c], Joanna Wietrzyk [c], Andrzej Katrusiak [b], Adam Huczyńsk i[a, *]

[a] *Department of Bioorganic Chemistry, Faculty of Chemistry, Adam Mickiewicz University, Uniwersytetu Poznańskiego 8, 61-614 Poznan, Poland.*

[b] *Department of Materials Chemistry, Faculty of Chemistry Adam Mickiewicz University, Uniwersytetu Poznańskiego 8, 61-614 Poznań, Poland*

[c] *Hirszfeld Institute of Immunology and Experimental Therapy, Polish Academy of Sciences, Rudolfa Weigla 12, 53-114 Wrocław, Poland*

Supplementary materials

Index

General procedures 1

The antiproliferative activity 2

X-ray measurements 4

Synthesis and characterization of 10-demethylcolchicine (colchiceine, **1**) 7

Synthesis and characterization of thiocolchicine 7

Synthesis and characterization of 1-demethylthiocolchicine (**3**) 7

^{1}H, ^{13}C NMR and ESI-MS spectra of compounds **2a-f** and **4a-f** 8

General procedures

All precursors for the synthesis of colchicine derivatives and solvents were obtained from Merck or Fluka and were used as received without further purification. $CDCl_3$ and DMSO-d_6 spectral–grade solvents were stored over 3 Å molecular sieves for several days. All the solvents used in flash chromatography were of HPLC grade (CHROMASOLV from Merck) and were used as received. Reaction mixtures were stirred using Teflon-coated magnetic stir bars. Reactions were monitored by thin layer chromatography (TLC) using aluminum-backed plates (Merck 60F254). TLC plates were visualized in UV-light (254 nm). The ^{1}H, ^{13}C spectra were recorded on a Varian VNMR-S 400 MHz spectrometer using TMS as the internal standard in both cases. No window function or zero filling was used. ^{1}H NMR measurements of 1-14 (0.07 mol dm^{-3}) in $CDCl_3$ were carried out at the operating frequency 402.64 MHz.

The error of the chemical shift value was 0.01 ppm. The ^{13}C NMR spectra were recorded at the operating frequency 101.25 MHz. The error of chemical shift value was 0.1 ppm. All spectra were locked to deuterium resonance of $CDCl_3$. The ESI (Electrospray Ionisation) mass spectra were recorded on a Waters/Micromass (Waters Corporation, Manchester, UK) ZQ mass spectrometer equipped with a Harvard Apparatus syringe pump. The samples were prepared in dry acetonitrile (5 x10-5 mol dm^{-3}). The sample was infused into the ESI source using a Harvard pump at a flow rate of 20 ml min-1. The ESI source potentials were: capillary 3 kV, lens 0.5 kV, extractor 4 V. The standard ESI mass spectra were recorded at the cone voltages: 10 and 30 V. The source temperature was 120 °C and the desolvation temperature was 300 °C. Nitrogen was used as the nebulizing and desolvation gas at flow-rates of 100 dm^3 h^{-1}. Mass spectra were acquired in the positive ion detection mode with unit mass resolution at a step of 1 m/z unit. The mass range for ESI experiments was from m/z = 100 to m/z = 1000, as well as from m/z = 200 to m/z = 1500

The antiproliferative activity

Four human cancer cell lines and one murine normal cell line were used to evaluate antiproliferative activity of colchicine and its derivatives: human lung adenocarcinoma (A549), human breast adenocarcinoma (MCF-7), human colon adenocarcinoma cell lines sensitive and resistant to doxorubicin (LoVo), respectively, and normal murine embryonic fibroblast cell line (BALB/3T3). The BALB/3T3 cell line was purchased from the American Type Culture Collection (ATCC, Manassas, VA, USA), A549 and MCF-7 cell lines—from European Collection of Authenticated Cell Cultures (Salisbury, UK), LoVo cell line was purchased from the ATCC (ATCC, Manassas, VA, USA). All the cell lines are maintained in the Institute of Immunology and Experimental Therapy (IIET), Wroclaw, Poland. Human lung adenocarcinoma cell line was cultured in mixture of OptiMEM and RPMI 1640 (1:1) medium (IIET, Wroclaw, Poland), supplemented with 5% fetal bovine serum (GE Healthcare, Logan, UT, USA) and 2 mM L-glutamine (Sigma-Aldrich, Merck KGaA, Saint Louis, MO, USA). Human breast adenocarcinoma cell line was cultured in mixture of Eagle medium (IIET, Wroclaw, Poland), supplemented with 10% fetal bovine serum, 2 mM L-glutamine, 8 μg/mL insulin and 1% amino-acids (Sigma-Aldrich, Merck KGaA, Saint Louis, MO, USA). Human colon adenocarcinoma cell lines were cultured in mixture of OptiMEM and RPMI 1640 (1:1) medium (IIET, Wroclaw, Poland), supplemented with 5% fetal bovine serum (GE Healthcare, Logan UT, USA), 2 mM L-glutamine, 1 mM sodium pyruvate (Sigma-Aldrich, Merck KGaA, Saint Louis, MO, USA). Murine embryonic fibroblast cells were cultured in

Dulbecco medium (Life Technologies Limited, Paisley, UK), supplemented with 10% fetal bovine serum (GE Healthcare, Logan, UT, USA) and 2 mM glutamine (Sigma-Aldrich, Merck KGaA, Saint Louis, MO, USA). All culture media contained antibiotics: 100 U/mL penicillin and 100 μg/mL streptomycin (Polfa-Tarchomin, Warsaw, Poland). All cell lines were cultured during entire experiment in humid atmosphere at 37 °C and 5% CO_2. Cells were tested for mycoplasma contamination by mycoplasma detection kit for conventional PCR: Venor GeM Classic (Minerva Biolabs GmbH, Berlin, Germany) and negative results was obtained. The procedure is repeated every year or in the case of less frequently used lines: after thawing.

The Antiproliferative Assays *In Vitro*

Twenty-four hours before adding the tested compounds, all cell lines were seeded in 384-well plates (Sarstedt, Nümbrecht, Germany) in appropriate media with $1x10^3$ or $1,5x10^3$ or $3x10^3$ cells per well. All cell lines were exposed to each tested agent at four different concentrations in the range 100–0.001 μg/mL for 72 h. Cells were also exposed to the reference drug cisplatin (Teva Pharmaceuticals Polska, Warsaw, Poland) and doxorubicin (Accord Healthcare Limited, Middlesex, UK). Additionally, all cell lines were exposed to DMSO (solvent used for tested compounds) (POCh, Gliwice, Poland) at concentrations corresponding to those present in tested agents' dilutions. After 72 h sulforhodamine B assay (SRB) was performed[1].

SRB

After 72 h of incubation with the tested compounds, cells were fixed in situ by gently adding of 30 μL per well of cold 50% trichloroacetic acid TCA (POCh, Gliwice, Poland) and were incubated at 4 °C for one hour. Following, wells were washed four times with water and air dried. Next, 25 μL of 0.1% solution of sulforhodamine B (Sigma-Aldrich, Merck KGaA, Saint Louis, MO, USA) in 1% acetic acid (POCh, Gliwice, Poland) were added to each well and plates were incubated at RT for 0.5 h. After incubation time, unbound dye was removed by washing plates four times with 1% acetic acid whereas stain bound to cells was solubilized with 10 mM Tris base (Sigma-Aldrich, Steinheim, Germany). Absorbance of each solution was read at Synergy H4 Hybrid Multi-Mode Microplate Reader (BioTek Instruments, Inc., Winooski, VT, USA) at the 540 nm wavelength.

Results are presented as mean IC_{50} (concentration of the tested compound, that inhibits cell proliferation by 50%) ± standard deviation. IC_{50} values were calculated in Cheburator 0.4, Dmitry Nevozhay software (version 1.2.0 software by Dmitry Nevozhay, 2004–

2014, **http://www.cheburator.nevozhay.com**, freely available) for each experiment[2]. Compounds at each concentration were tested in triplicates in single experiment and each experiment was repeated at least three times independently.

X-ray measurements

The X-ray diffraction measurements at RT of 3, 4a and 4e, were carried out on two different diffractometers. First one equipped with MoKα (λ =0.7108 Å) source, Eos Xcalibur, and the second one, with microfocus CuKα (λ =1.5418 Å) radiation source, SuperNova Agilent diffractometer. For data collections and their preliminary reduction, CrysAlisPro 171.37.31 software was used[3]. Crystal structure was solved by direct methods with Shelxs and further refined with the Shelxl[4,5]; with the Olex2 interface[6].The position of hydrogen atoms were located at their best geometrical positions. In the structure 4e disorder on C9 carbonyl group and C10 thiomethoxy group was refined with half occupancy in two positions accordingly. The details of crystal data, data collection, structure solution and refinement are given in Table S2. Details on data collection and refinement, fractional atomic coordinates, anisotropic displacement parameters and full list of bond lengths and angles in the crystallographic-information-file (CIF) format has been deposited with the Cambridge Crystallographic Database Centre as a supplementary Publication No. 1966195, 1966194 and 1966196 for compound **3**, **4a** and **4e** respectively. Copies of this information may be obtained free of charge from http://www.ccdc.cam.ac.uk

Table S1. Length and angle values of H-bonds present in molecules **3**, **4a** and **4e**. The asterisk marks the intermolecular H-bond.

Compound	H-bond	Length	Angle Value	Symmetric code
**3**	N–H···S	2.793(2) Å	137.77(1)°	-x, ½+y, -z
	N–H···O4	2.370(3) Å	159.17(3)°	-x, 1/2+y, -z
	O1–H···O4	1.974(4) Å	146.64(1)°	1+x, y, z
**4a**	N–H···OH_2	2.086(1) Å	167.13(2)°	x, y, z
	HO–H···O4	1.994(3) Å	151.10(5)°	x, 1+y, z
	HO–H···O5	2.007(3) Å	157.67(3)°	2-x, ½+y, -z
**4e**	N–H···O5	2.033(2) Å	158.76(1)°	-x, ½+y, -z
	HO–H···O4	2.555(1) Å	149.46(3)°	-x, -½+y, -z
	HO–H···O5	2.538(1) Å	146.10(2)°	x, y, z

Table S2. Detailed crystallographic data of **3**, **4a** and **4e**.

		3	4a	4e
Formula		$C_{21}H_{22}NO_5S$	$C_{23}H_{25}NO_6S \cdot H_2O$	$C_{23}H_{25}NO_7S \cdot ½H_2O$
Wavelength (Å)		0.71073	1.54184	1.54184
Crystal system		orthorhombic	monoclinic	monoclinic
Space group		$P2_12_12_1$	$P2_1$	$P2_1$
Unit cell dimensions	*a* (Å)	9.1005(17)	10.600(2)	10.8835(10)
	b(Å)	11.866(2)	6.9635(11)	9.2158(6)
	c (Å)	17.881(4)	16.712(3)	12.0632(12)
	α (°)	90	90	90
	β (°)	90	107.07(2)	104.613(9)
	γ (°)	90	90	90
Volume ($Å^3$)		1930.8(7)	1179.2(4)	1170.80(18)
Z/Z'		4/1	2/1	2/1
D_x (g/cm^3)		1.378	1.300	1.329
Absorption (mm^{-1})		0.201	1.587	1.624
F(000)		844.0	488.0	494.0
Crystal size (mm)		0.403 × 0.155 × 0.038	0.636 × 0.141 × 0.089	0.517 × 0.173 × 0.05
2*θ*-range (°)		5.642 to 52.742	11.926 to 152.97	7.574 to 152.97
Min/max indices: h, k, l		$-10 \leq h \leq 11$, $-14 \leq k \leq 14$, $-21 \leq l \leq 22$	$-10 \leq h \leq 10$, $-6 \leq k \leq 6$, $-16 \leq l \leq 16$	$-13 \leq h \leq 12$, $-11 \leq k \leq 6$, $-15 \leq l \leq 14$
Ref. collected/unique		3875/2540	4653/1011	4646/3124
R_{int}		0.1442	0.0811	0.0757
Completeness (%)		100%	93%	92%
Data/restrains/ parameter		3875/126/258	1011/277/300	3124/129/357
Goodness-of-fit on F2		0.889	1.028	1.014
Final R1/wR2 (I>2σ1)		0.0789/0.0835	0.0887/0.2070	0.0575/0.1551
R1/wR2 (all data)		0.2254/0.1254	0.1045/0.2270	0.0731/0.1724
Largest diff. peak/hole ($e.Å^{-3}$)		0.23/-0.26	0.26/-0.29	0.23/-0.21

Synthesis and characterization of 10-demethylcolchicine (colchiceine, 1)

A mixture of colchicine (500 mg, 1.25 mmol), glacial acetic acid (0.5 mL), and hydrochloric acid (0.1N, 30 mL) was stirred for 6h at 100 °C. After that time crystals start to precipitate, and X-ray analysis, which was in consistence with results obtained by Mackey et al.[33], confirmed them to be the desired products. **1** was obtained as yellow-brown crystals (435 mg, 90%) and directly used for next steps.

^{1}H NMR ($CDCl_3$, 400 MHz) δ 7.65 (1H, s), 7.59 (1H, d, J = 11.5 Hz), 7.49 (1H, s), 7.34 (1H, d, J = 11.5 Hz), 6.57 (1H, s), 4.71 – 4.64 (1H, m), 3.94 (3H, s), 3.91 (3H, s), 3.64 (3H, s), 2.53 – 2.48 (1H, m), 2.36 – 2.27 (2H, m), 2.00 (3H, s), 1.97 – 1.89 (1H, m). ^{13}C NMR ($CDCl_3$, 400 MHz) δ 170.13, 170.07, 169.9, 153.6, 151.5, 150.8, 141.5, 141.3, 136.3, 134.3, 125.9, 122.2, 119.4, 107.3, 61.3, 61.25, 56.0, 52.7, 37.4, 29.7, 22.7. ESI-MS (*m/z*): $[M+Na]^+$ 408, $[M+K]^+$ 424, $[2M+Na]^+$ 793.

Synthesis and characterization of thiocolchicine

To a mixture of colchicine (500 mg, 1.25 mmol) in MeOH/water (1/1, v/v, 5 mL), the sodium methanethiolate (solution 21% in H_2O, 0.83 mL, 2.5 mmol) was added. The mixture was stirred in at RT for 24 h. Reaction was controlled by TLC. After that time, the reaction mixture was quenched by the addition of water (150 mL). The whole mixture was extracted four times with CH_2Cl_2, and the combined organic layers were dried over $MgSO_4$, filtered, and evaporated under reduced pressure. The residue was purified by CombiFlash® (hexane/EtOAc (1/1), then EtOAc/MeOH, increasing concentration gradient) to give thiocolchicine as amorphous yellow solid with yield 78% . ^{1}H, ^{13}C NMR and ESI-MS analysis were in agreement with the one obtained by Shi *et al*[7].

^{1}H NMR ($CDCl_3$, 400 MHz) δ 7.92 (1H, s), 7.46 (1H, s), 7.36 – 7.26 (1H, m), 7.10 (1H, d, J = 10.5 Hz), 6.55 (1H, s), 4.73 – 4.63 (1H, m), 3.95 (3H, s), 3.91 (3H, s), 3.67 (3H, s), 2.54 (1H, dd, J = 13.0, 5.8 Hz), 2.45 (3H, s), 2.33 (2H, dtd, J = 31.1, 12.8, 6.5 Hz), 1.99 (3H, s), 1.96 – 1.85 (1H, m). ^{13}C NMR ($CDCl_3$, 400 MHz) δ 182.4, 170.0, 158.1, 153.6, 151.8, 151.1, 141.6, 138.6, 134.8, 134.4, 128.3, 126.7, 125.6, 107.3, 61.6, 61.4, 56.1, 52.3, 36.4, 29.9, 22.8, 15.1. ESI-MS (*m/z*): [M+H]+ 416, $[M+Na]^+$ 438, $[M+K]^+$ 454, $[2M+Na]^+$ 853, $[3M+Na]^+$ 1268.

Synthesis and characterization of 1-demethylthiocolchicine (3)

To the solution of thiocolchicine (1.0 g, 2.41 mmol) in dichloromethane (DCM, 50 ml) cooled to the 0 °C temperature, an acetyl chloride (3 mL) and tin tetrachloride (1mL) were added. The mixture was first stirred at 0 °C temperature for 1h, and then for the next 48h at RT. After that time TLC analysis showed no presence of thiocolchicine, and the DCM was evaporated to dryness under reduced pressure. The residue was dissolved in MeOH/water (5/1, v/v, 60 mL), then lithium hydroxide (excess) was added and the mixture was stirred at RT for 1h. Reaction time was determined by TLC. The solvent was evaporated to dryness under reduced pressure and the residue was purified by CombiFlash® (chloroform/acetone, increasing concentration gradient) to give product as amorphous yellow solid with 40% yield.

^{1}H NMR (DMSO-*d6*, 400 MHz) δ 9.09 (1H, s), 8.52 (1H, d, J = 7.8 Hz), 7.29 (1H, d, J = 10.4 Hz), 7.24 (1H, d, J = 10.9 Hz), 7.04 (1H, s), 6.50 (1H, s), 3.83 (3H, s), 3.74 (3H, s), 2.58 – 2.53 (1H, m), 2.43 – 2.39 (3H, m), 2.26 – 2.15 (1H, m), 2.09 – 1.99 (1H, m), 1.86 (3H, s), 1.84 – 1.77 (1H, m). ^{13}C NMR (DMSO-d_6, 101 MHz) δ 181.1, 168.3, 156.6, 152.5, 151.3, 147.0, 138.2, 135.2, 134.4, 134.2, 128.1, 126.7, 119.2, 103.2, 60.4, 55.7, 50.8, 36.4, 29.5, 22.6, 14.4. ESI-MS (*m/z*): $[M+H]^+$ 402, $[M+Na]^+$ 424, $[M+K]^+$ 440, $[2M+Na]^+$ 825.

^{1}H, ^{13}C NMR and ESI-MS spectra of compounds 2a-f and 4a-f

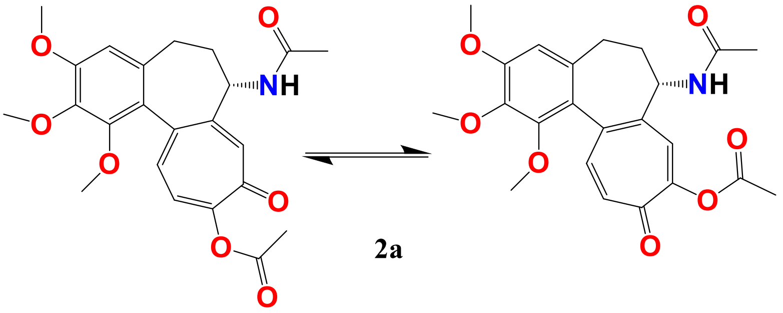

2a

Chemical formula: $C_{23}H_{25}NO_7$, MW = 427,5 g/mol

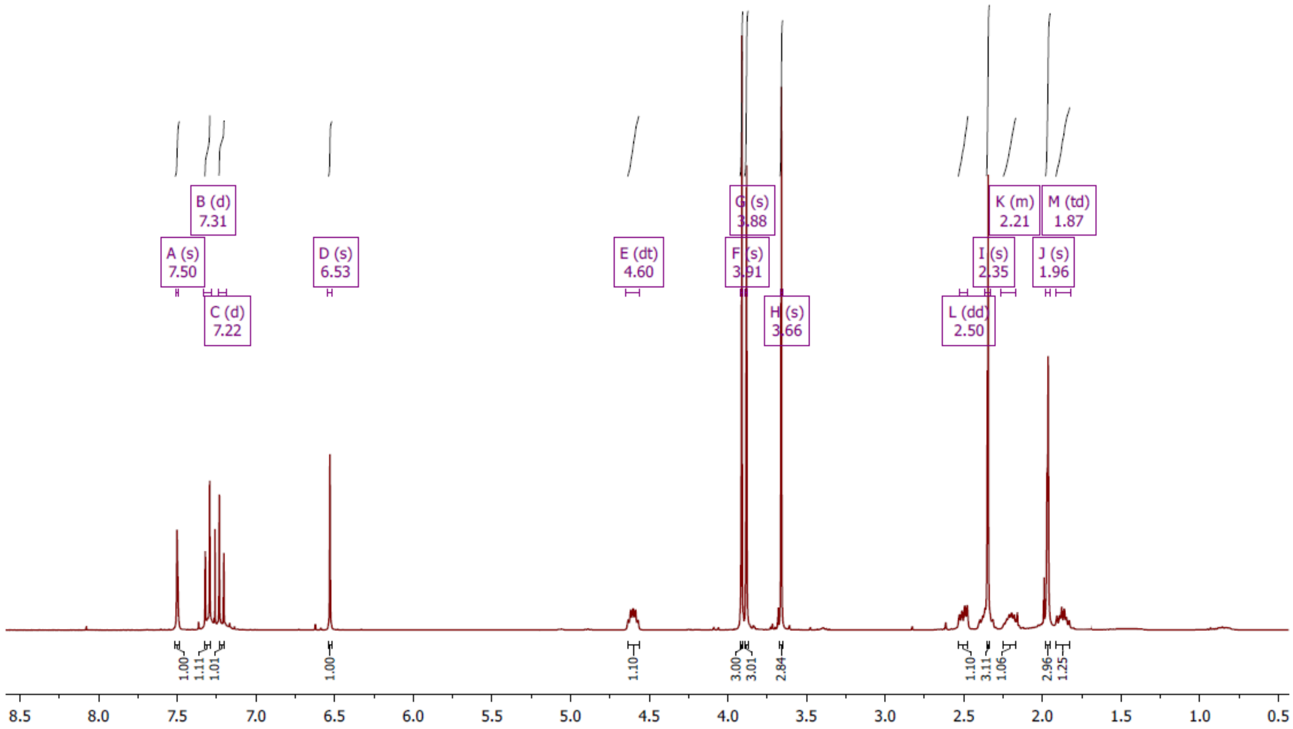

Figure S1. The ^{1}H NMR spectrum of **2a** in $CDCl_3$.

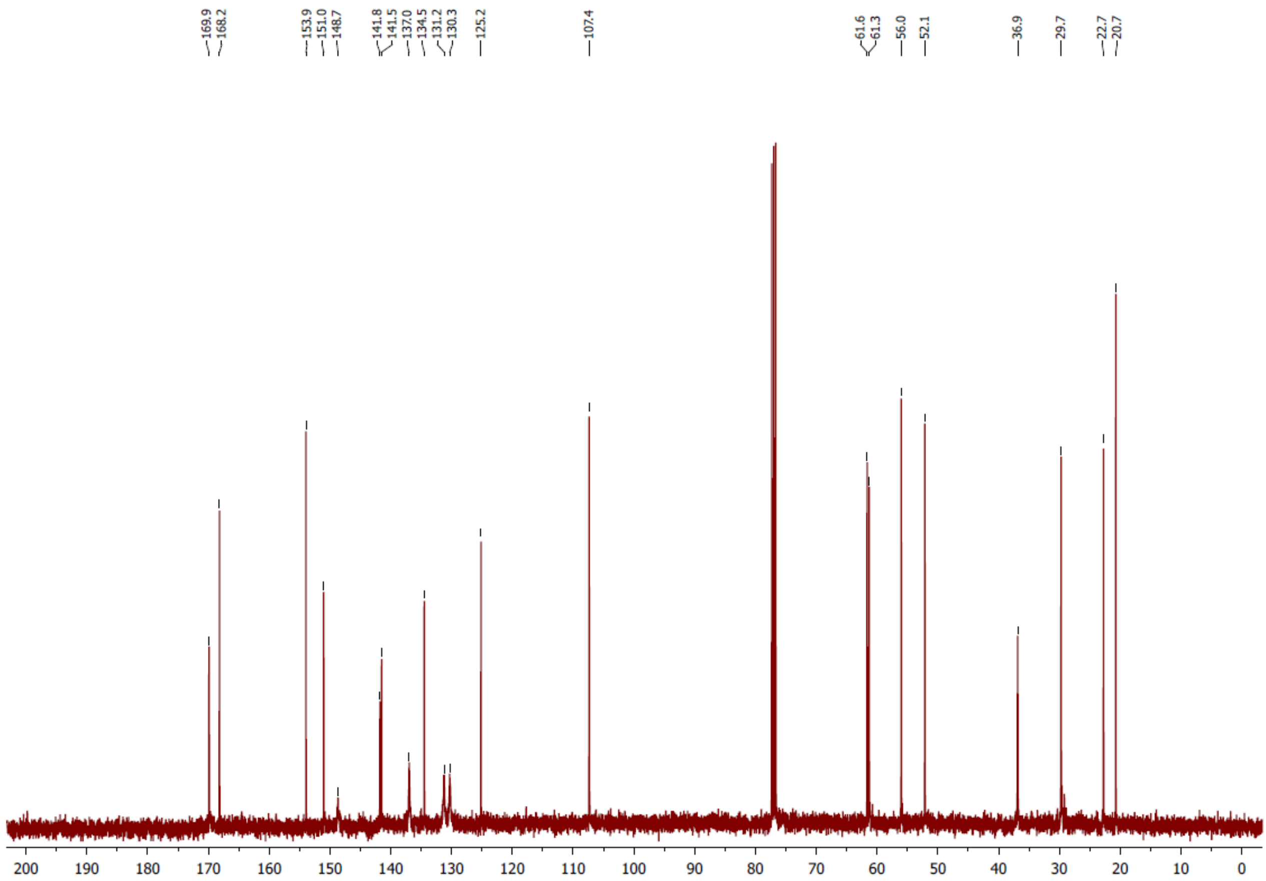

Figure S2. The ^{13}C NMR spectrum of **2a** in $CDCl_3$.

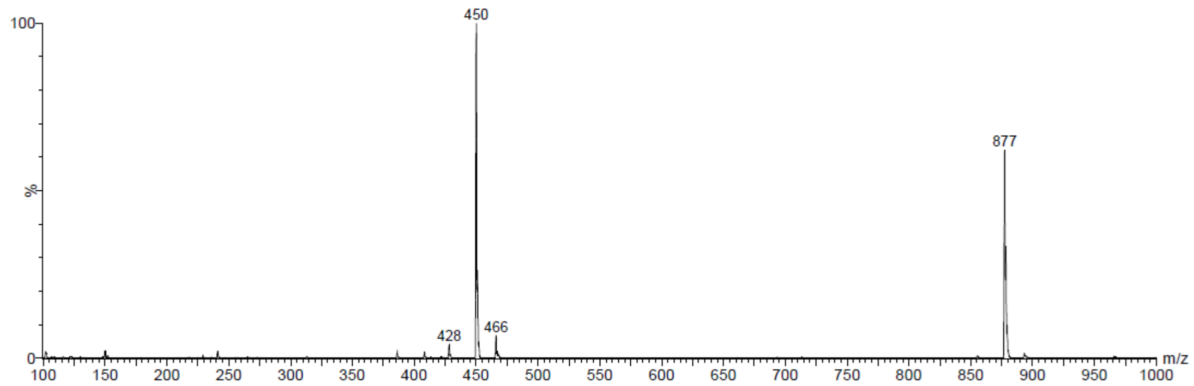

Figure S3. The ESI-MS spectrum of **2a**.

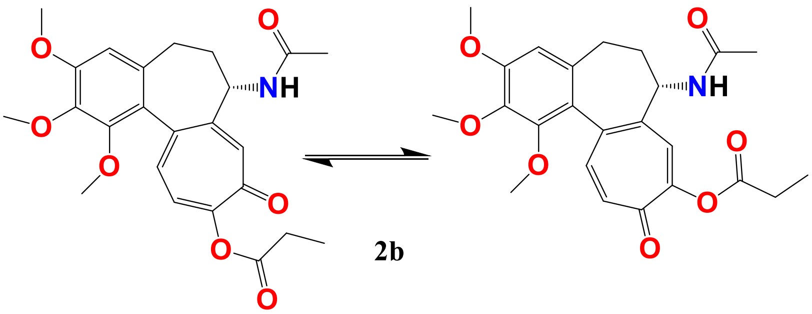

2b

Chemical formula: $C_{24}H_{27}NO_7$, MW = 441,5 g/mol

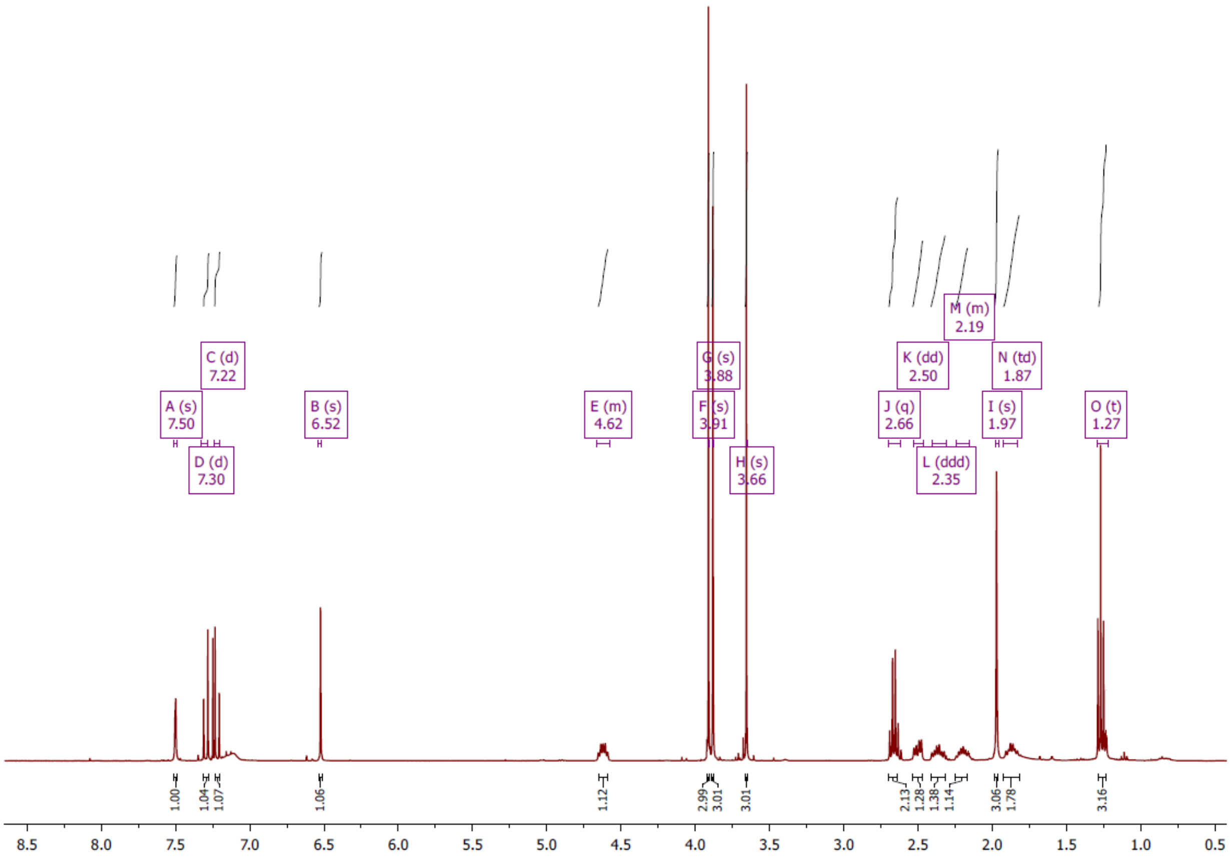

Figure S4. The ^{1}H NMR spectrum of **2b** in $CDCl_3$.

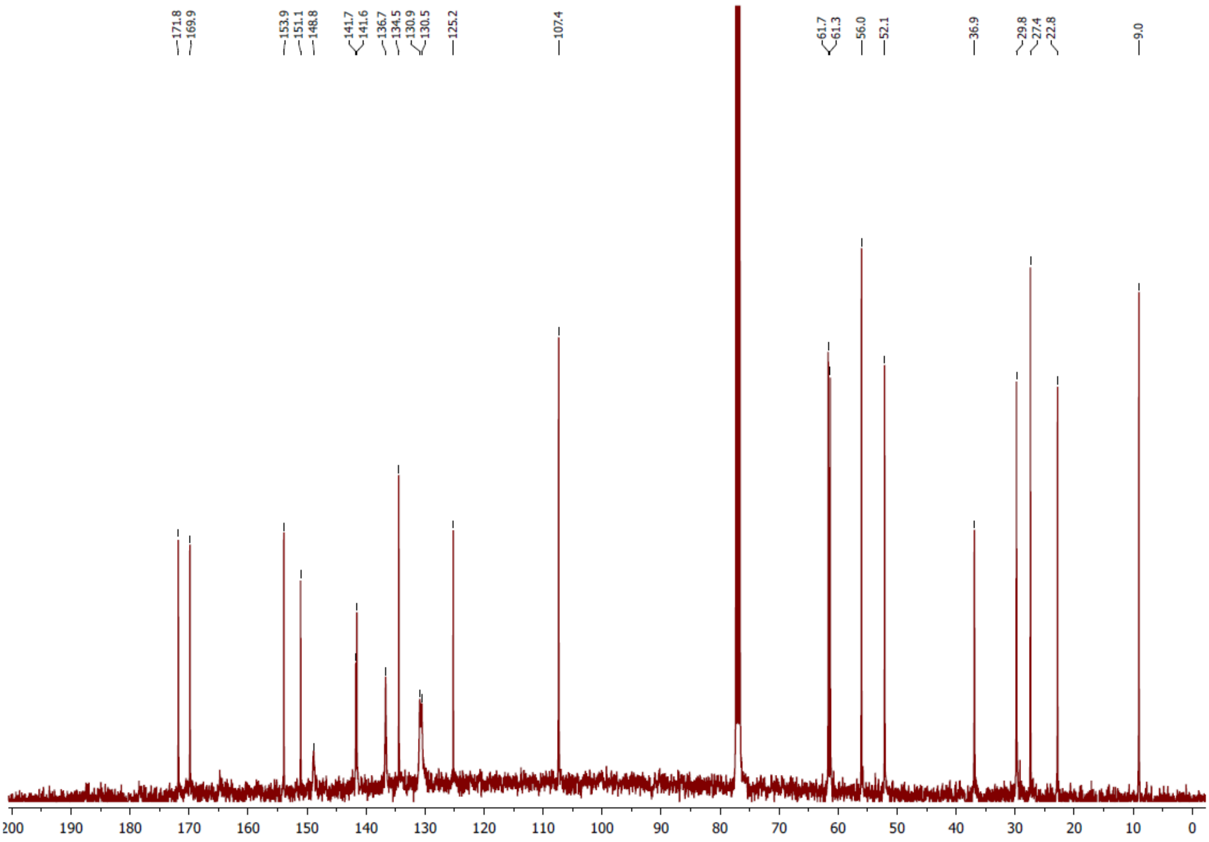

Figure S5. The ^{13}C NMR spectrum of **2b** in $CDCl_3$.

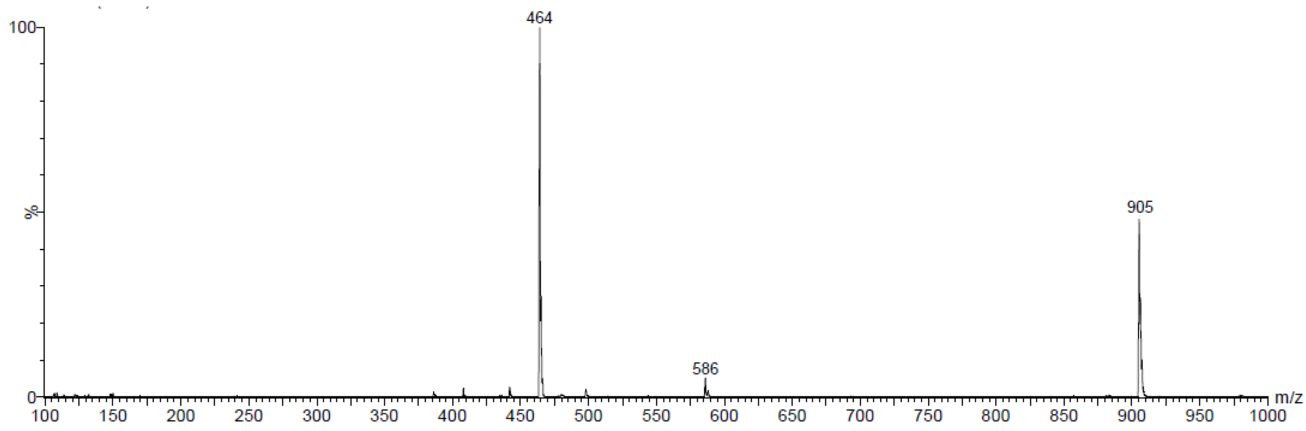

Figure S6. The ESI-MS spectrum of **2b**.

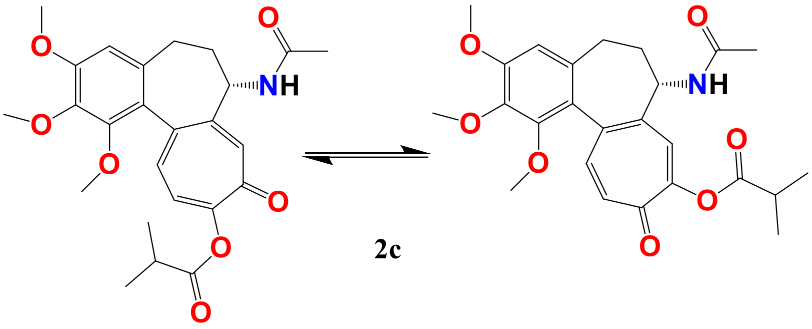

Chemical formula: $C_{25}H_{29}NO_7$, MW = 455,5 g/mol

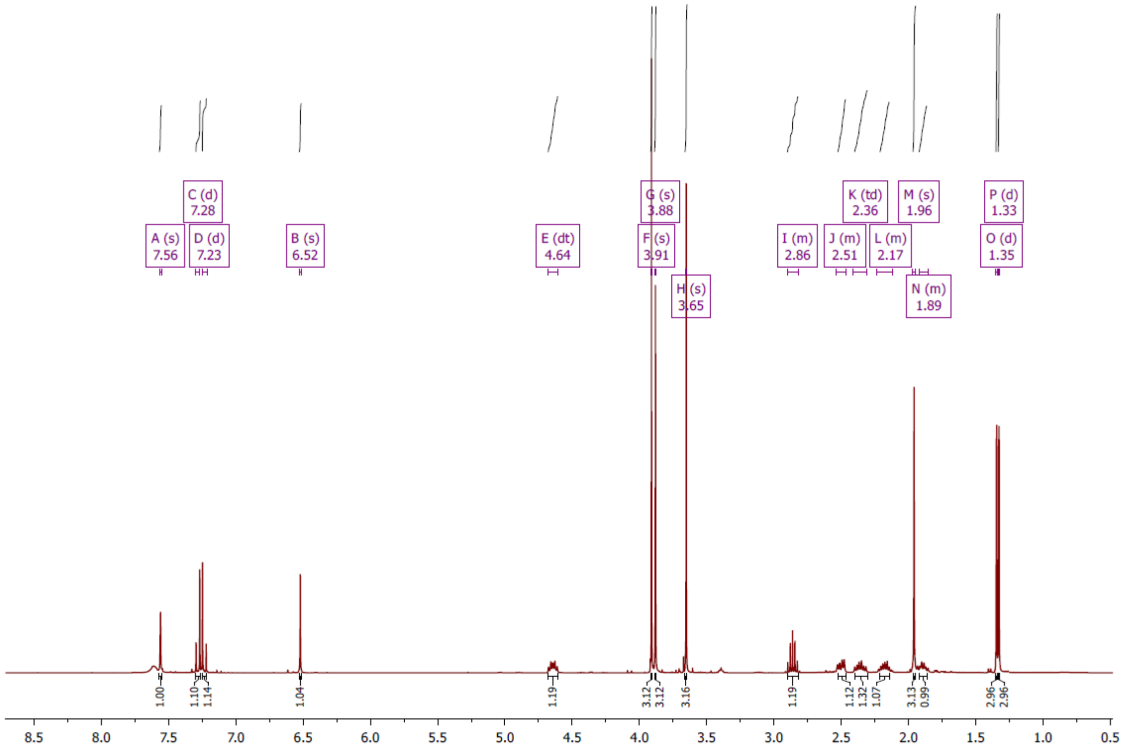

Figure S7. The ^{1}H NMR spectrum of **2c** in $CDCl_3$.

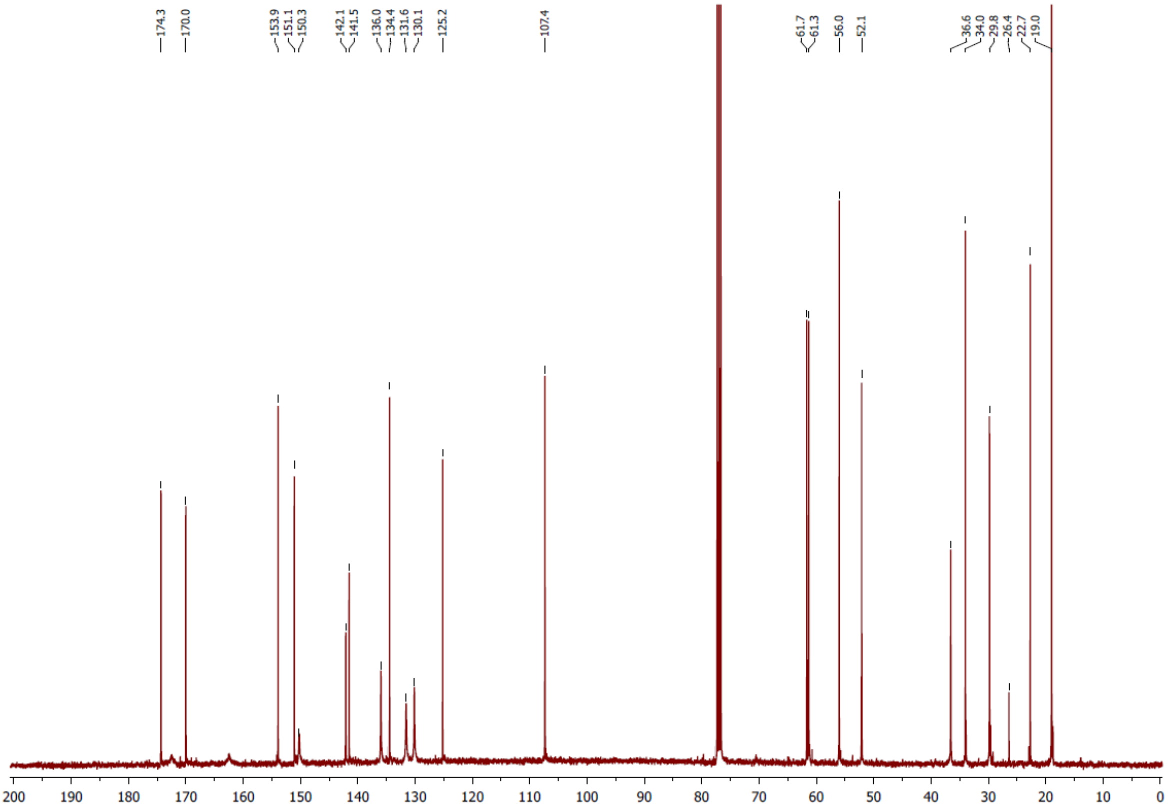

Figure S8. The ^{13}C NMR spectrum of **2c** in $CDCl_3$.

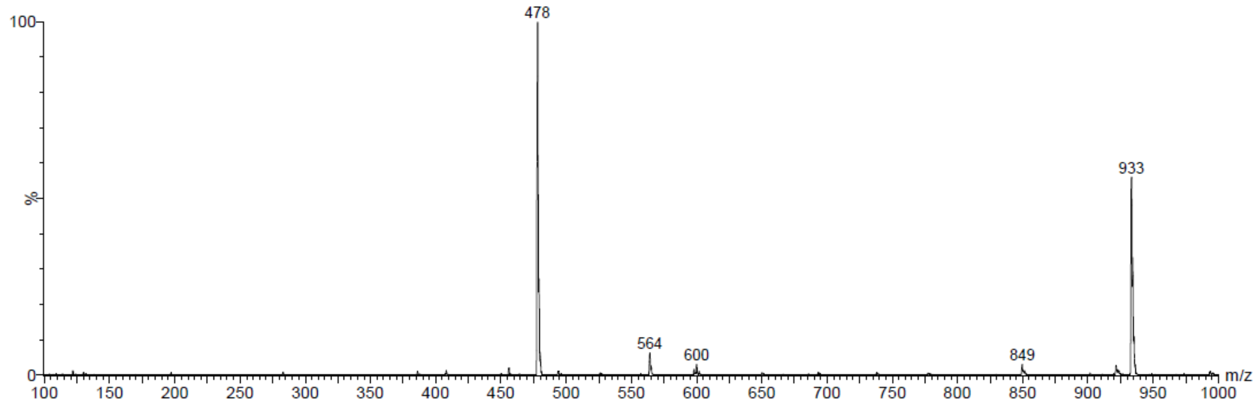

Figure S9. The ESI-MS spectrum of **2c**.

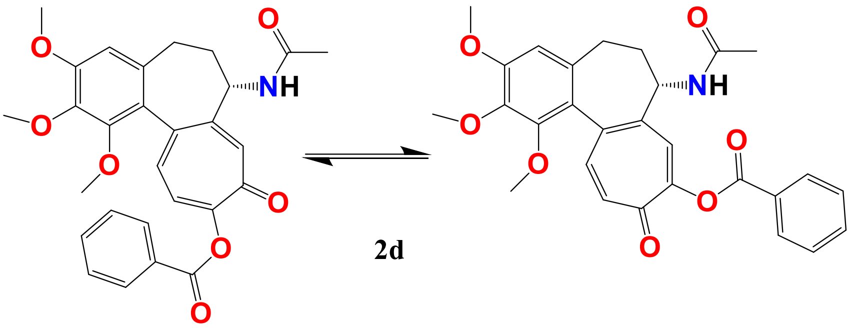

2d

Chemical formula: $C_{28}H_{27}NO_7$, MW = 489,5 g/mol

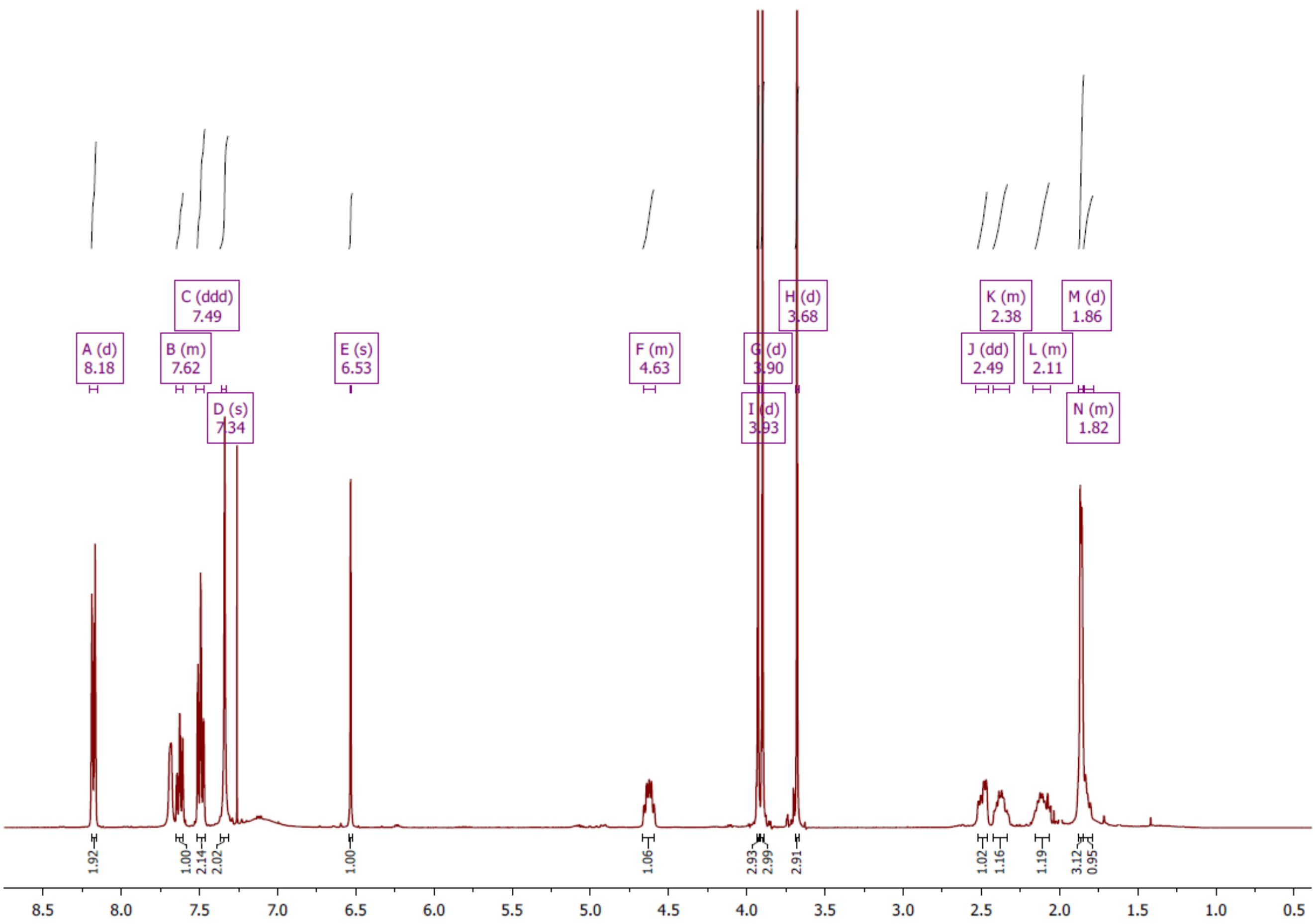

Figure S10. The ^{1}H NMR spectrum of **2d** in $CDCl_3$.

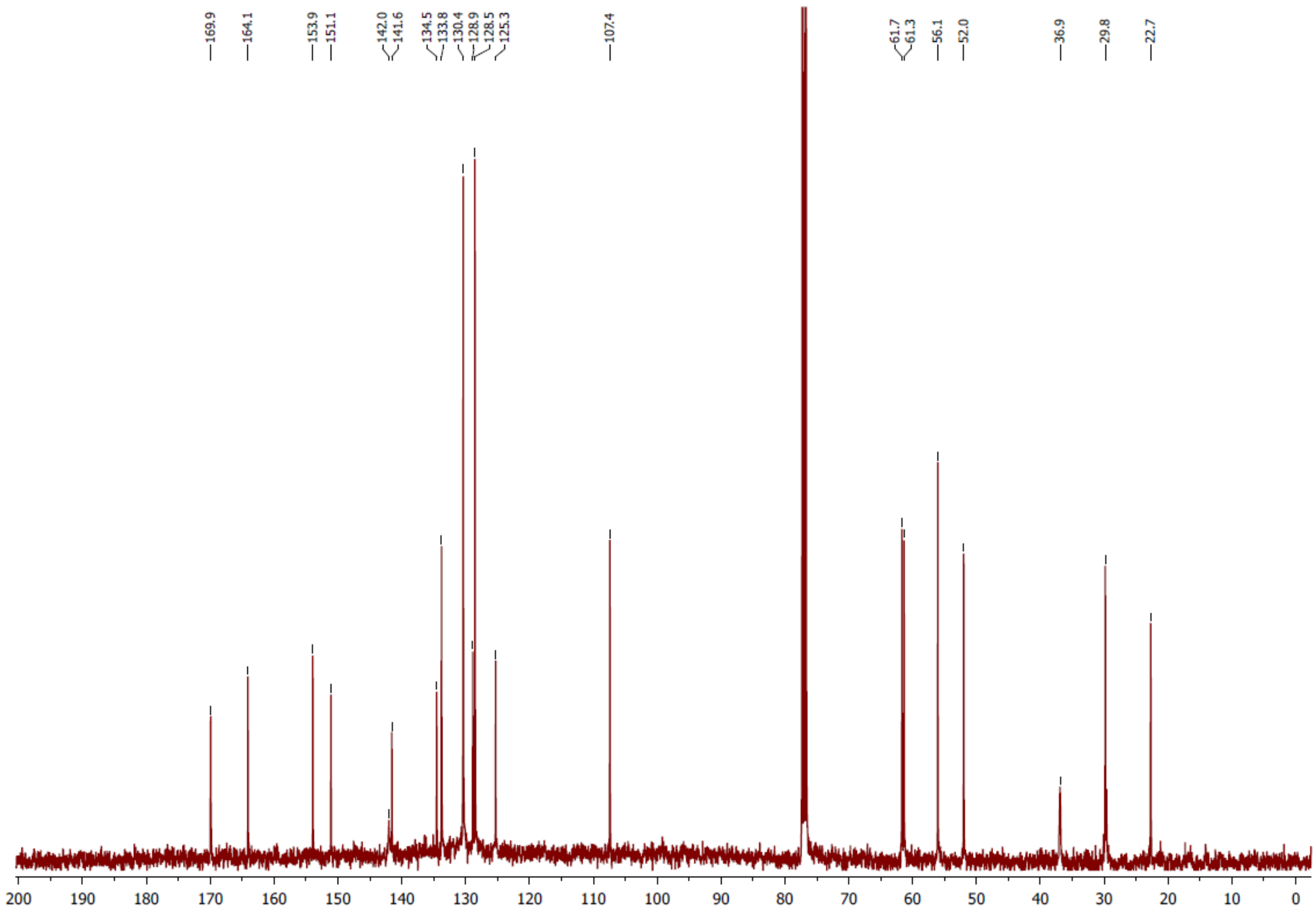

Figure S11. The ^{13}C NMR spectrum of **2d** in $CDCl_3$.

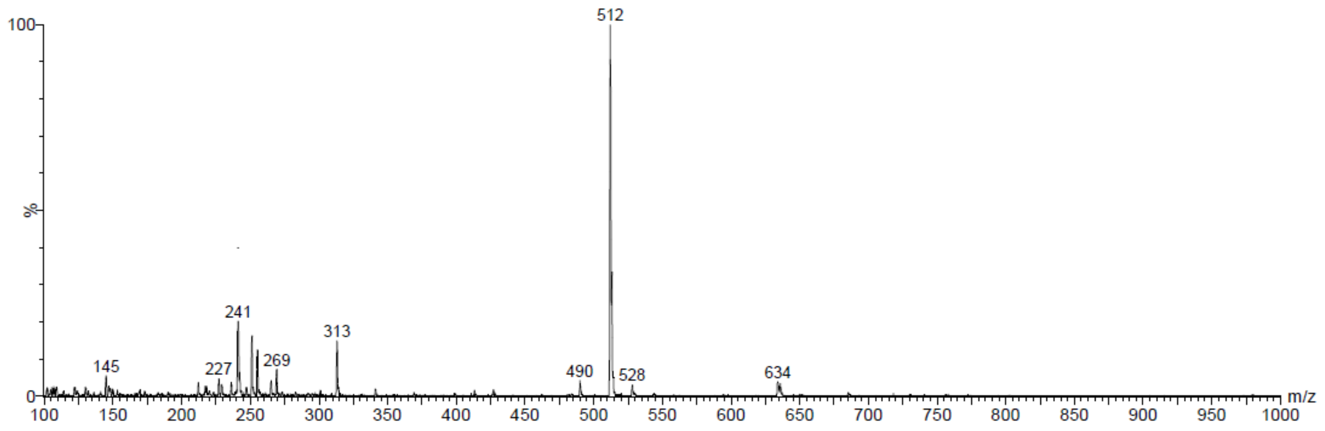

Figure S12. The ESI-MS spectrum of **2d**.

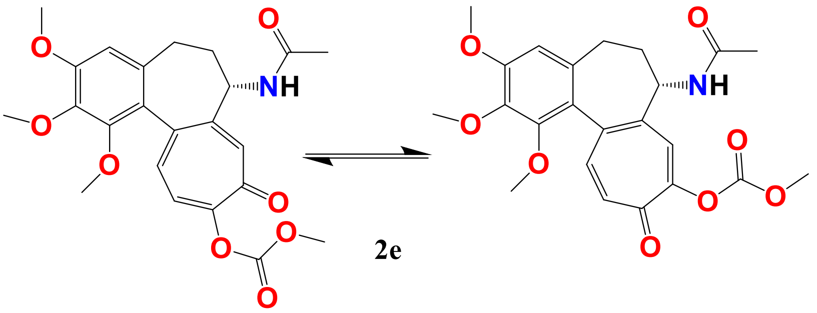

2e

Chemical formula: $C_{23}H_{25}NO_8$, MW = 443,5 g/mol

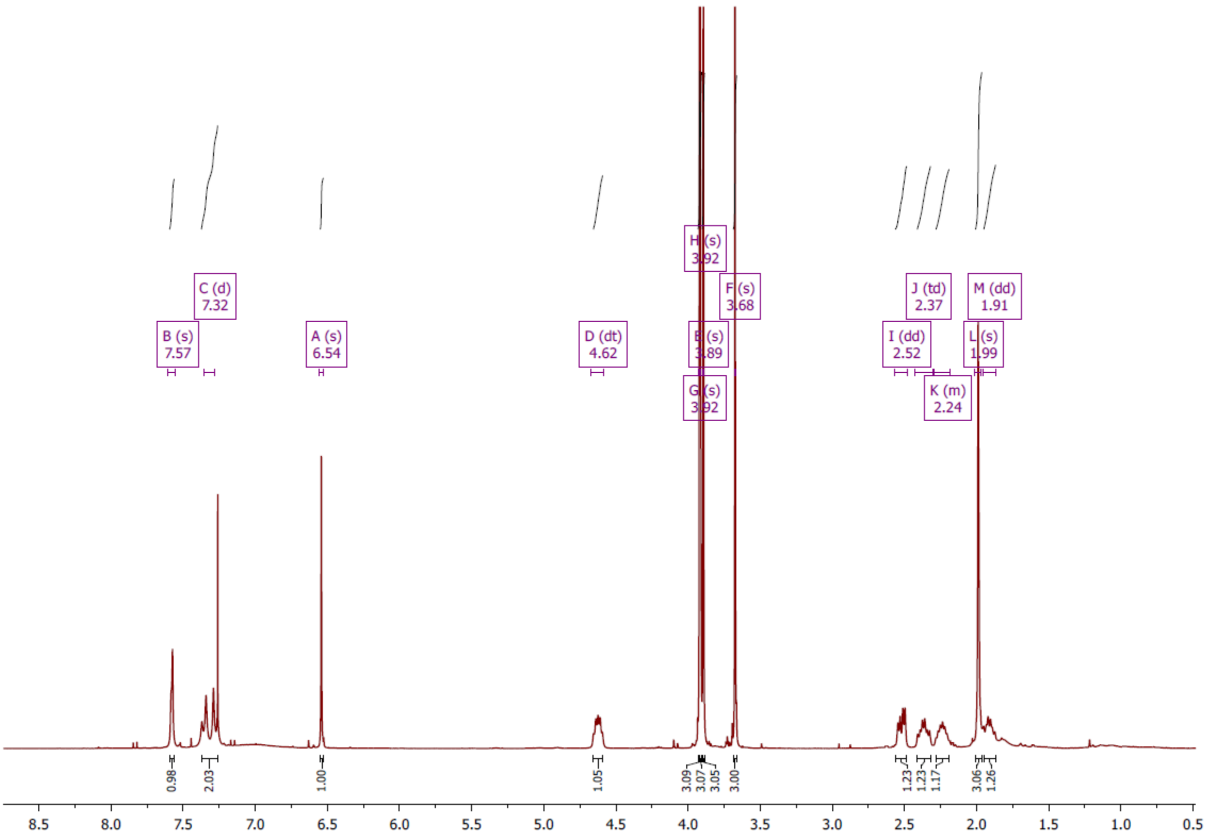

Figure S13. The ^{1}H NMR spectrum of **2e** in $CDCl_3$.

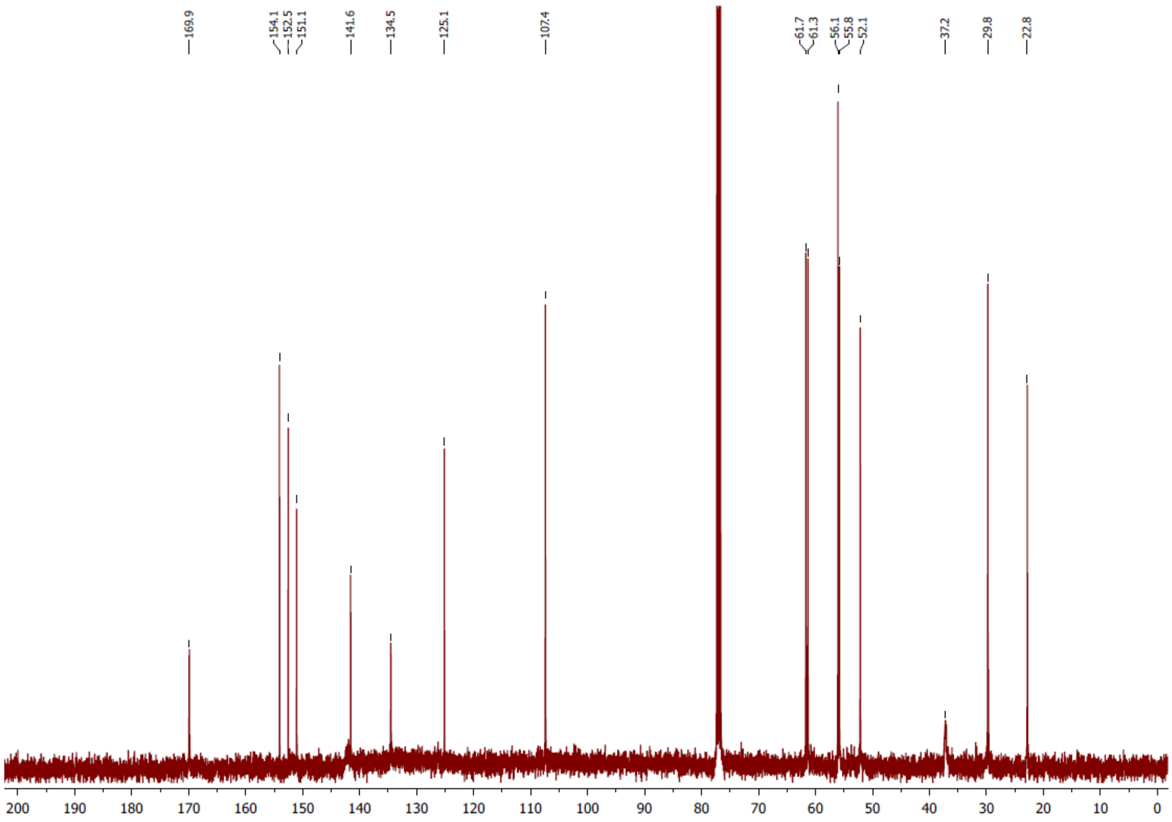

Figure S14. The ^{13}C NMR spectrum of **2e** in $CDCl_3$.

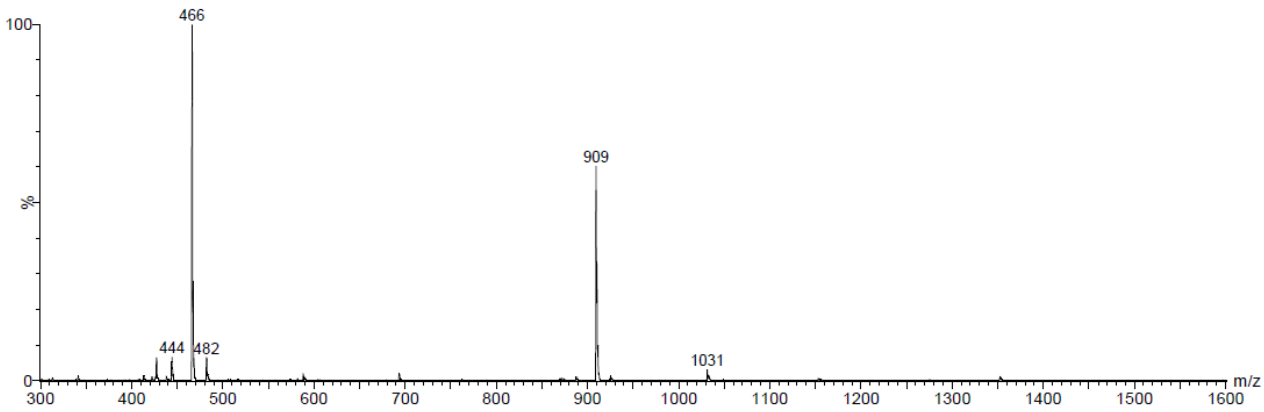

Figure S15. The ESI-MS spectrum of **2e**.

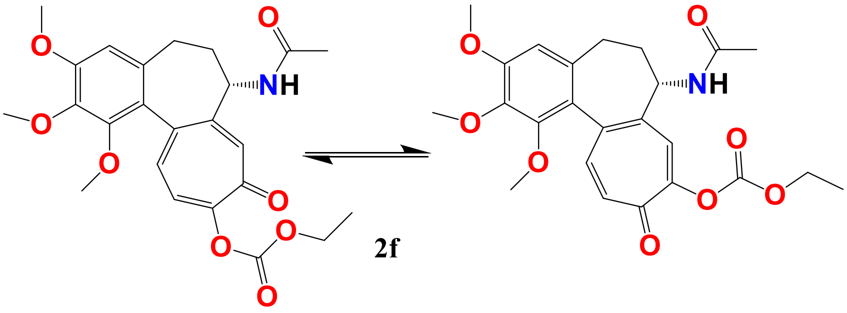

2f

Chemical formula: $C_{24}H_{27}NO_8$, MW = 457,5 g/mol

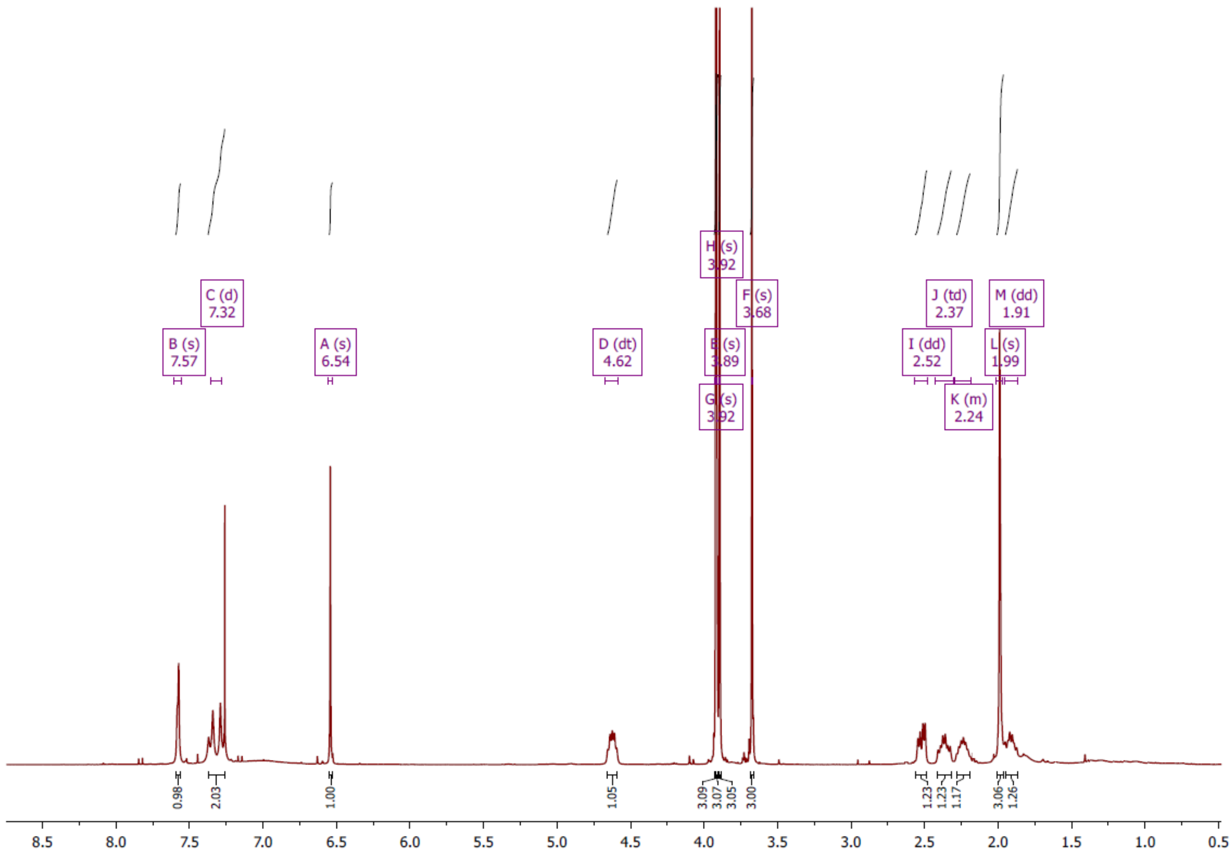

Figure S16. The ^{1}H NMR spectrum of **2f** in $CDCl_3$.

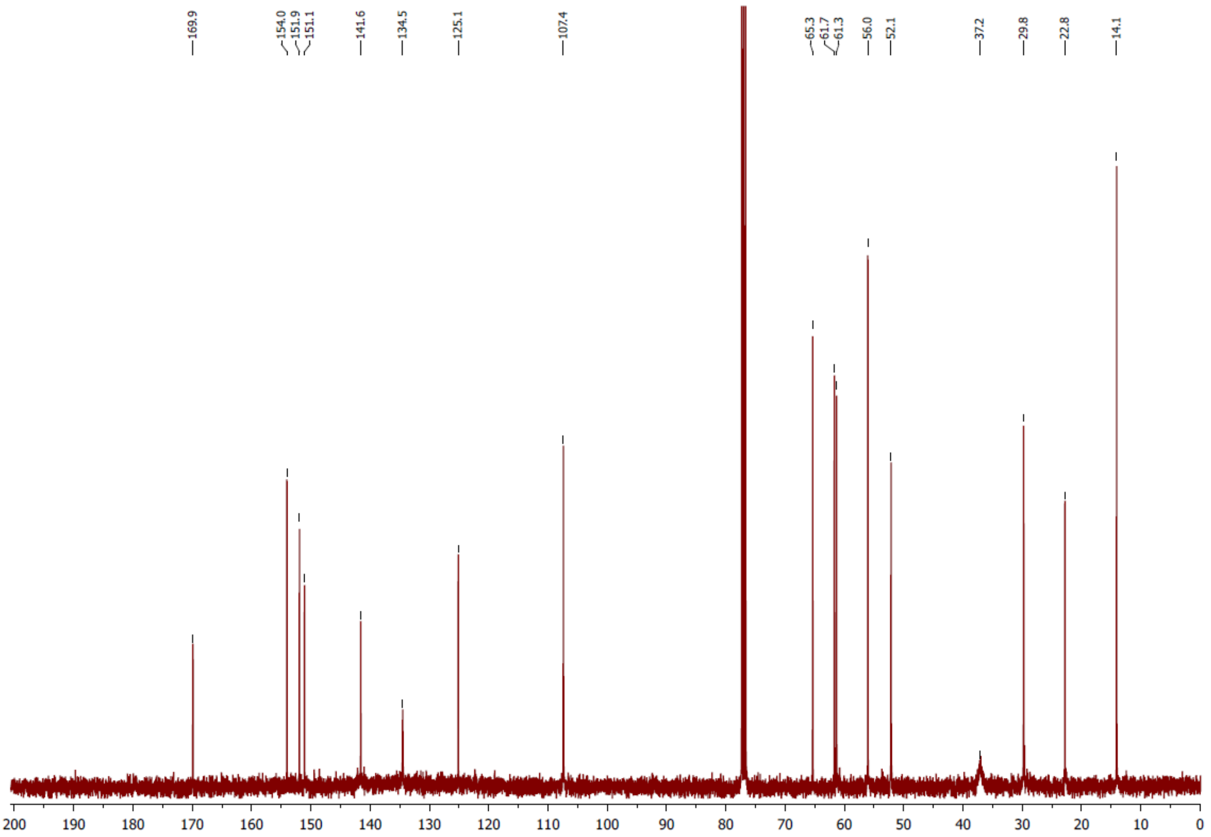

Figure S17. The ^{13}C NMR spectrum of **2f** in $CDCl_3$.

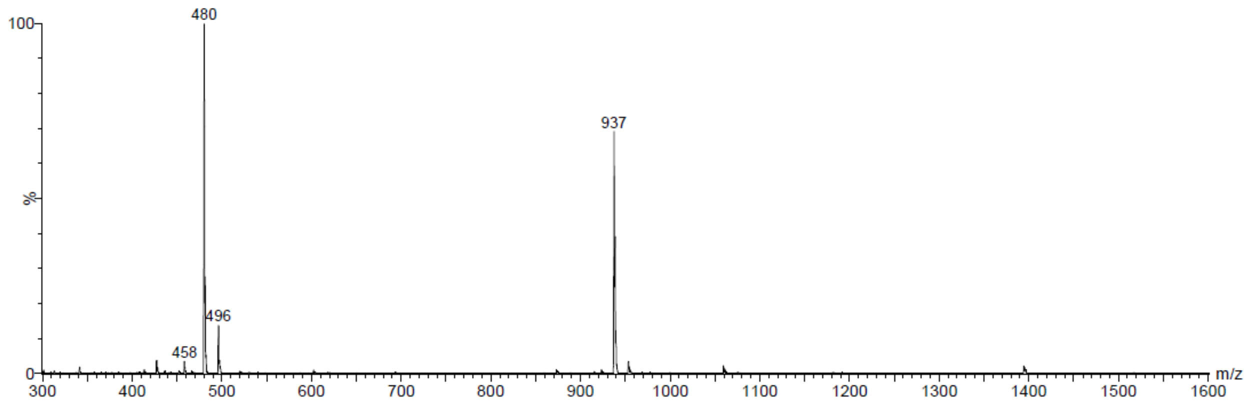

Figure S18. The ESI-MS spectrum of **2f**.

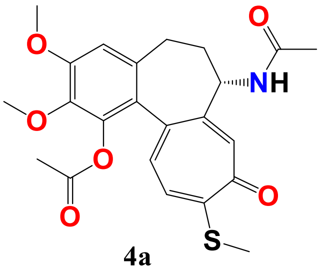

4a

Chemical formula: $C_{23}H_{25}NO_6S$, MW = 443,5 g/mol

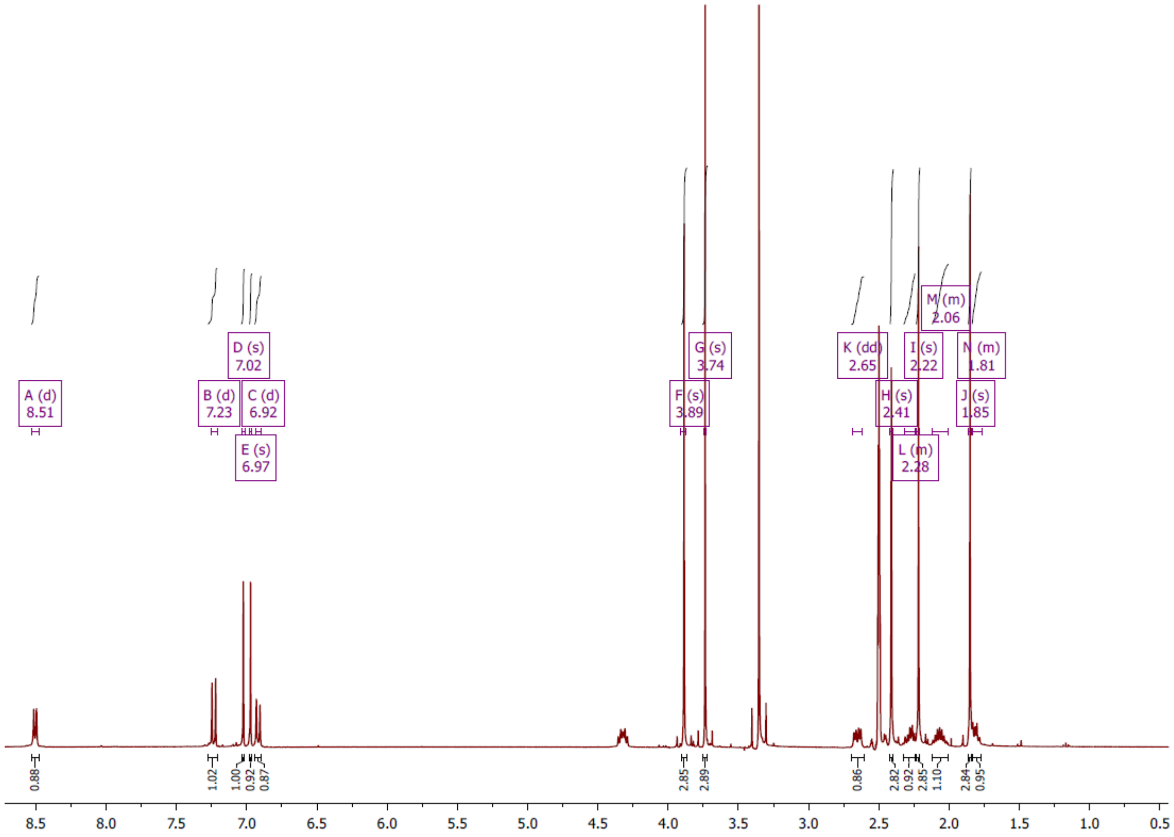

Figure S19. The ^{1}H NMR spectrum of **4a** in DMSO-d_6.

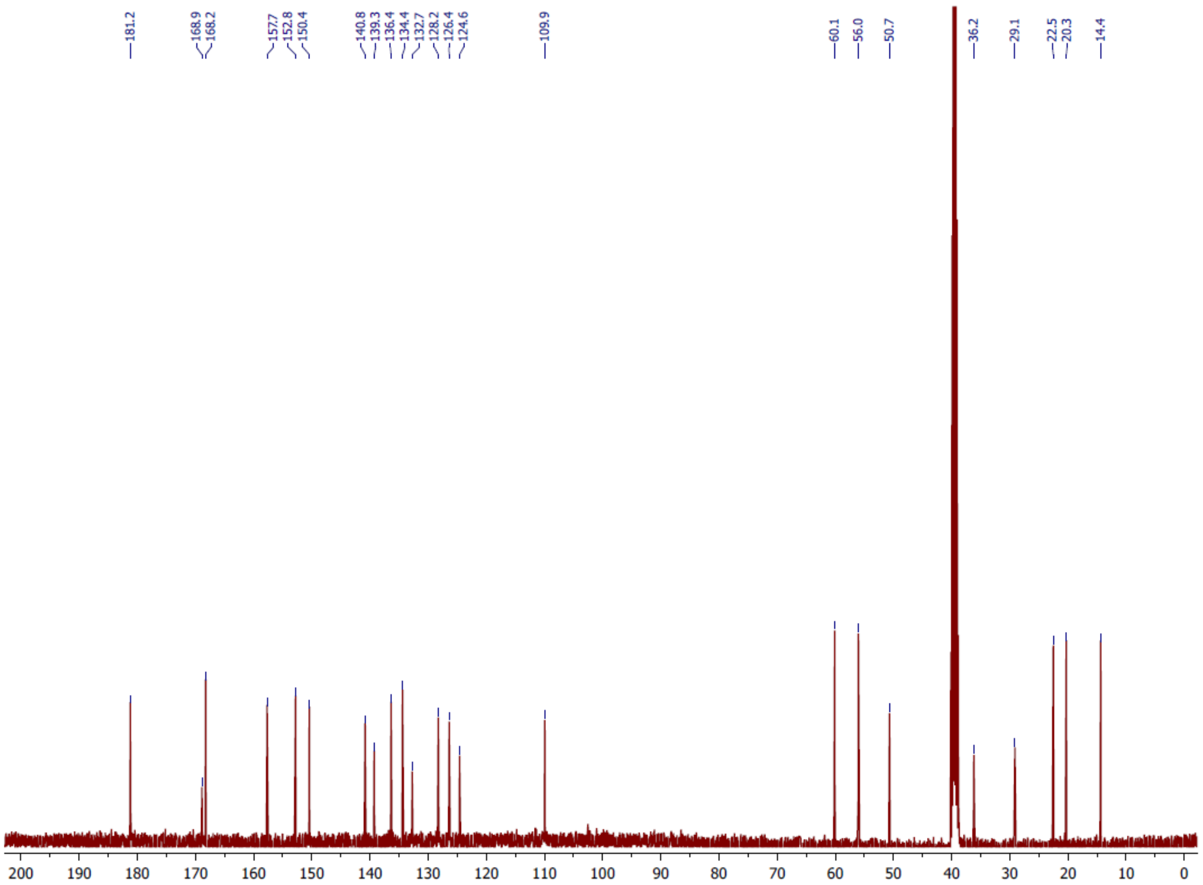

Figure S20. The ^{13}C NMR spectrum of **4a** in DMSO-d_6.

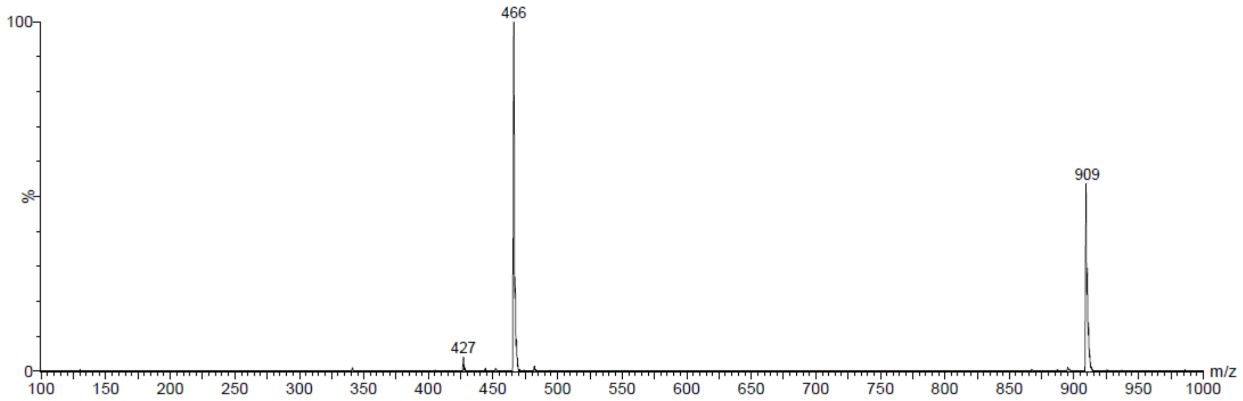

Figure S21. The ESI-MS spectrum of **4a**.

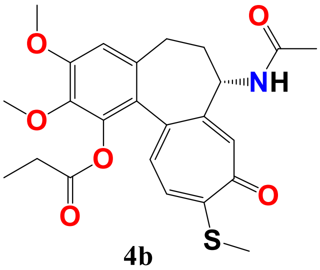

4b

Chemical formula: $C_{24}H_{27}NO_6S$, MW = 457,5 g/mol

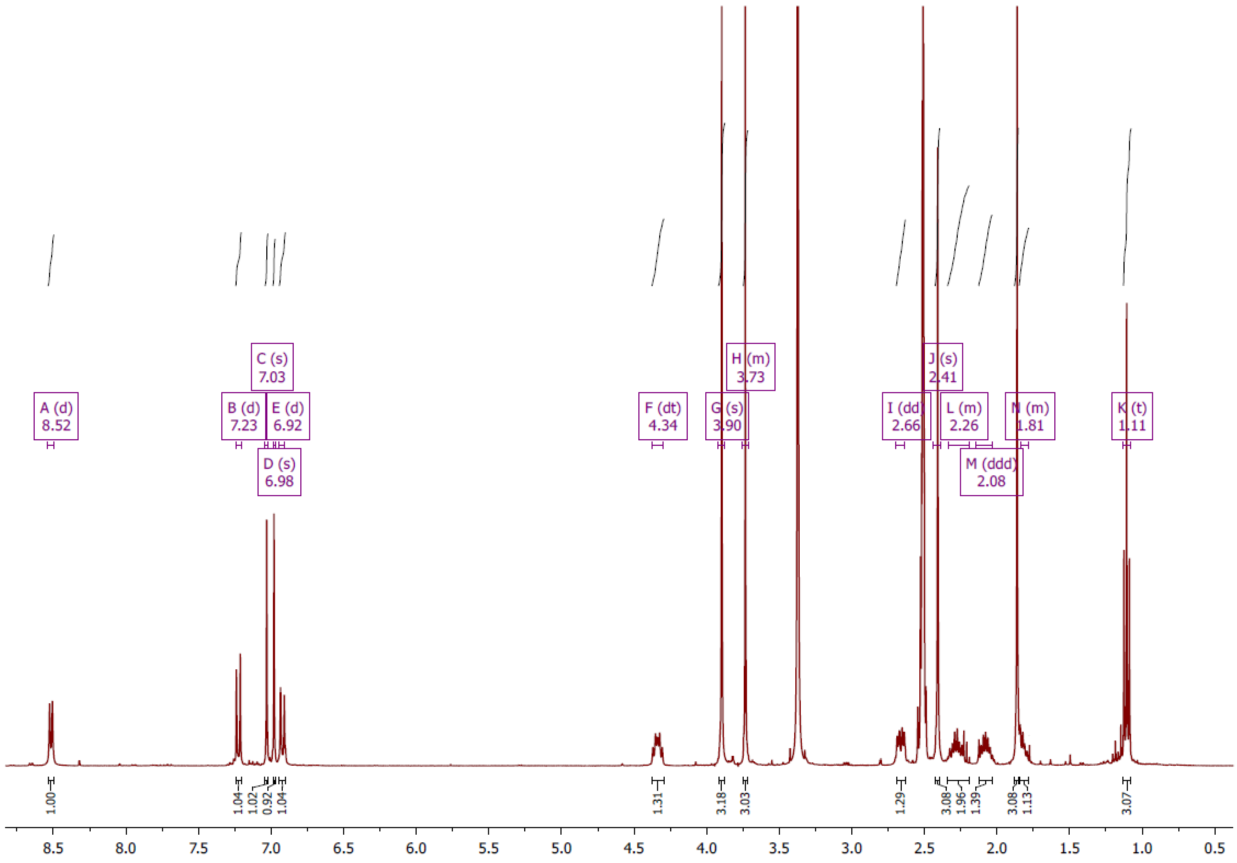

Figure S22. The ^{1}H NMR spectrum of **4b** in DMSO-d_6.

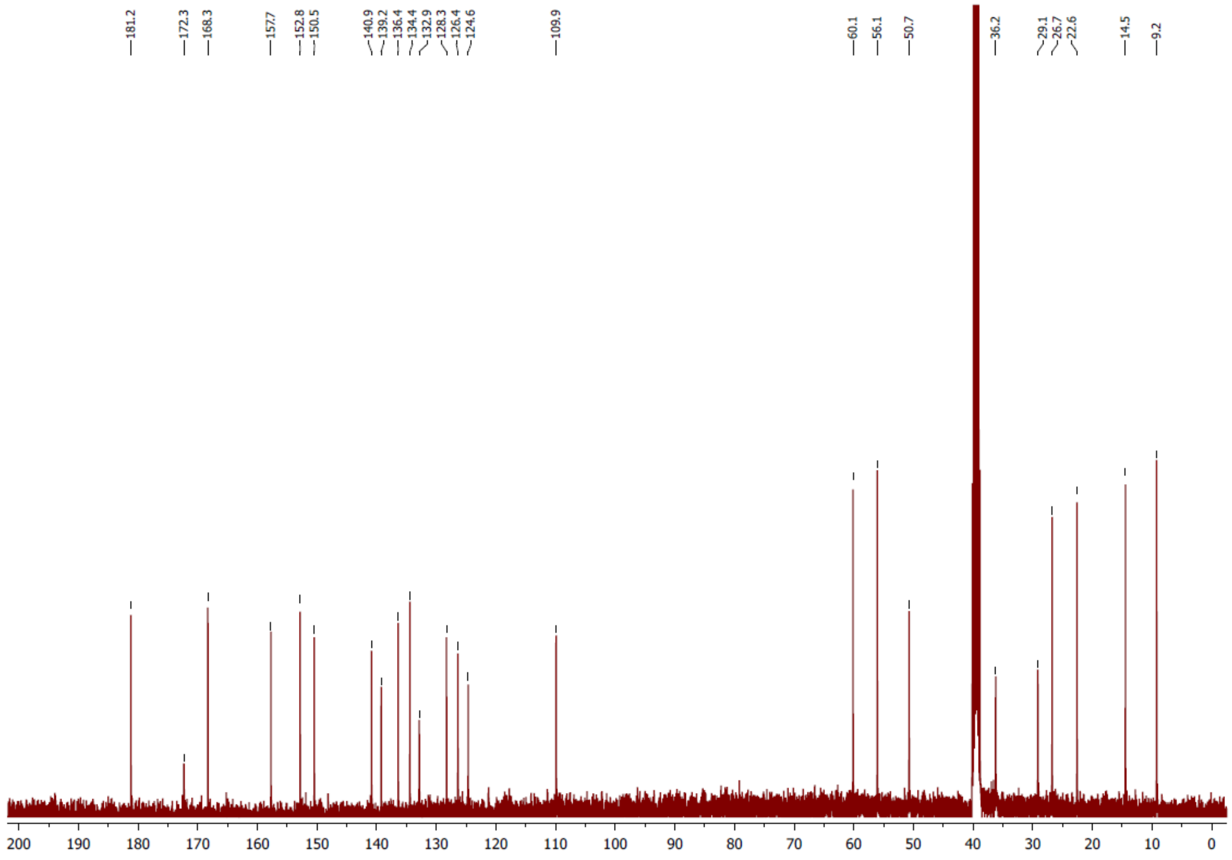

Figure S23. The ^{13}C NMR spectrum of **4b** in DMSO-d_6.

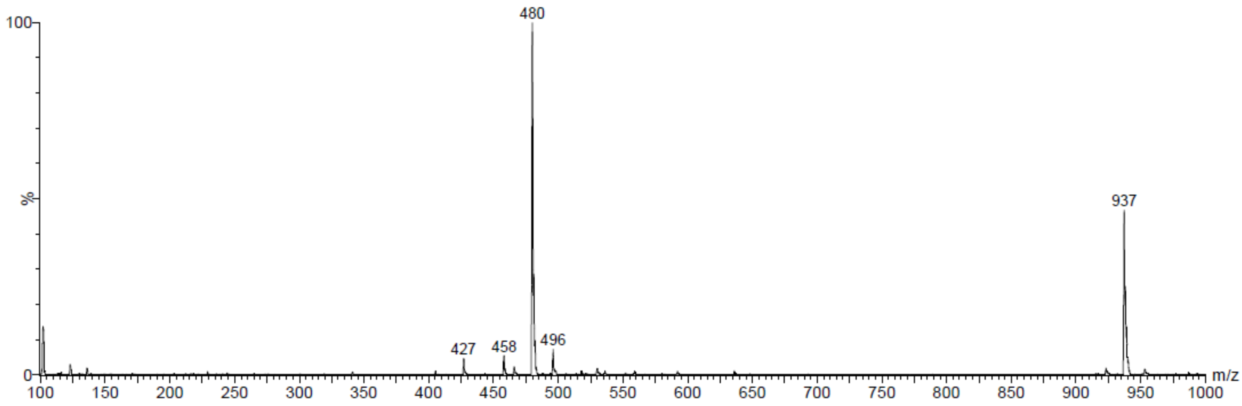

Figure S24. The ESI-MS spectrum of **4b**.

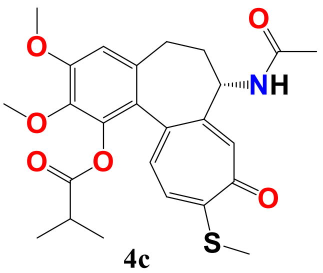

4c

Chemical formula: $C_{25}H_{29}NO_6S$, MW = 471,6 g/mol

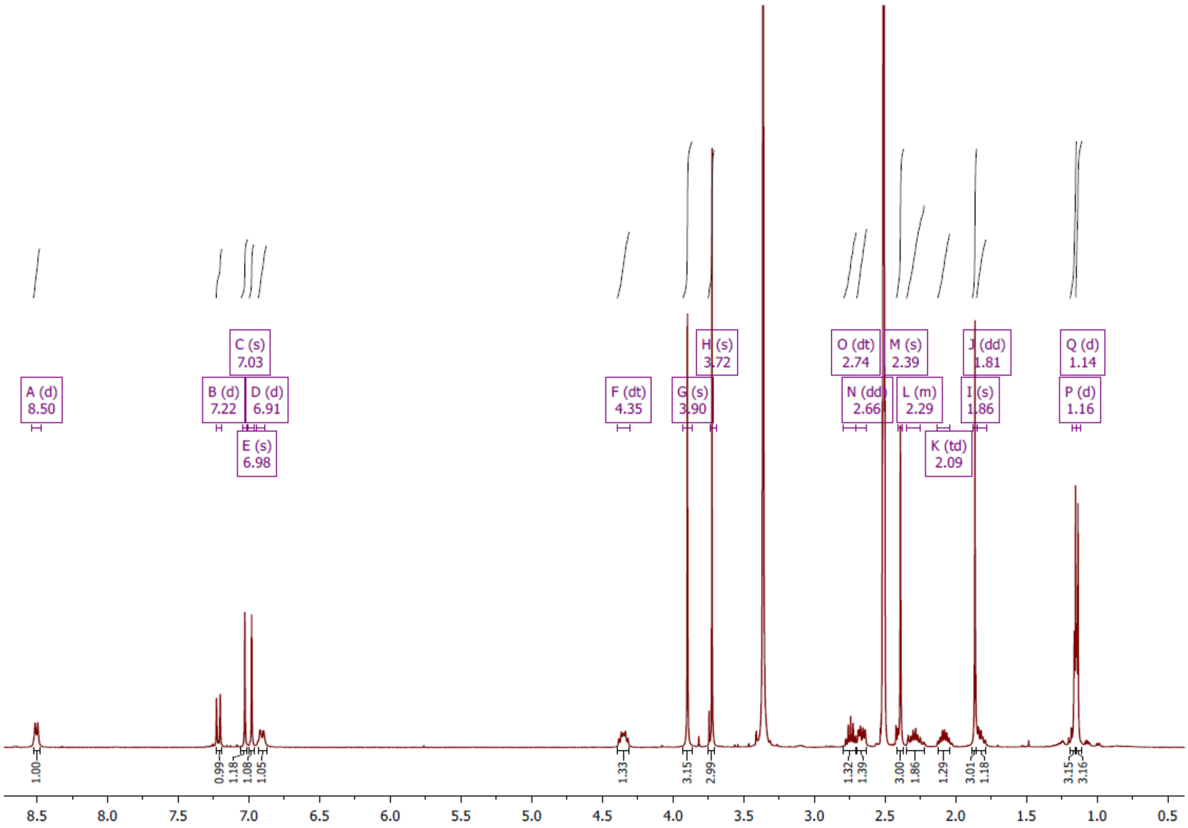

Figure S25. The ^{1}H NMR spectrum of **4c** in DMSO-d_6.

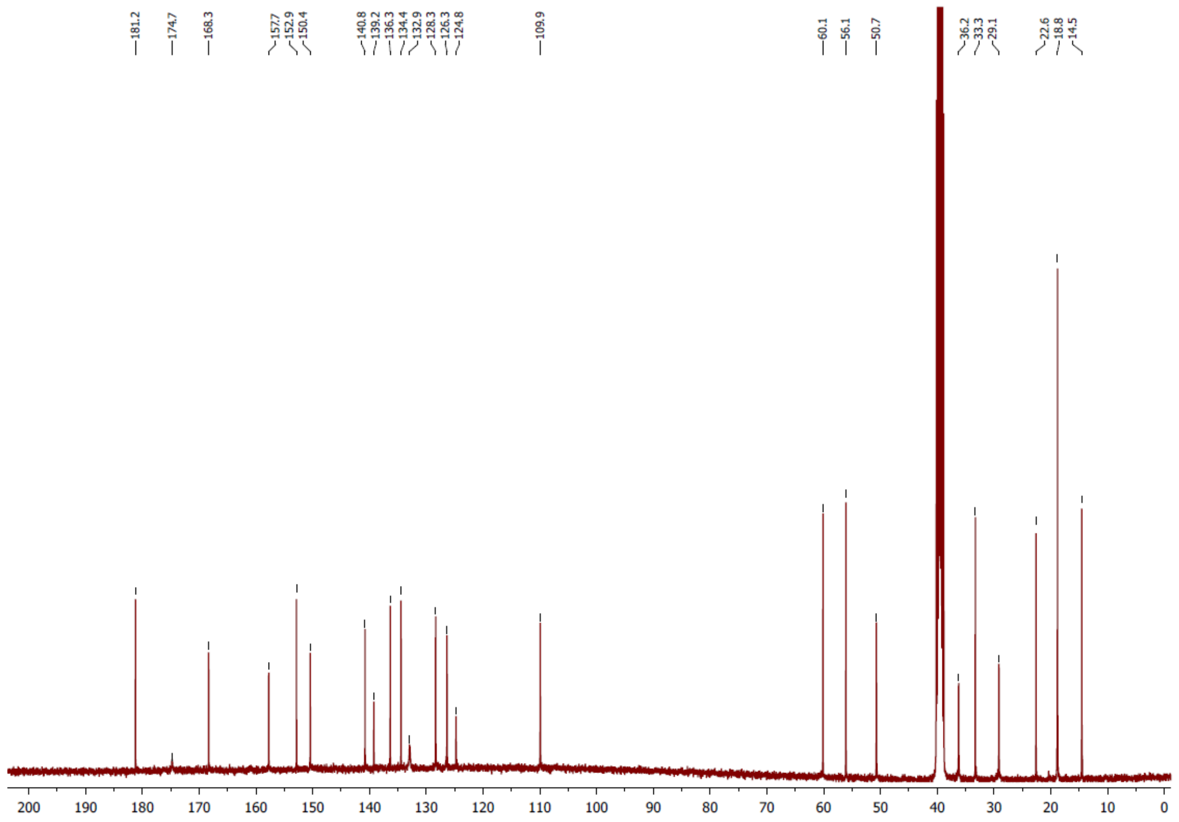

Figure S26. The ^{13}C NMR spectrum of **4c** in DMSO-d_6.

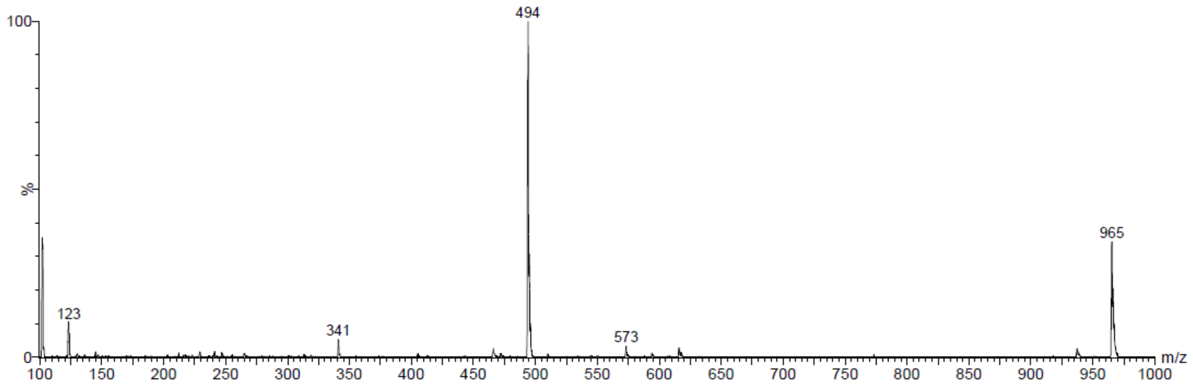

Figure S27. The ESI-MS spectrum of **4c**.

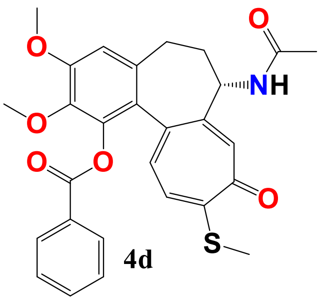

4d

Chemical formula: $C_{28}H_{27}NO_6S$, MW = 505,6 g/mol

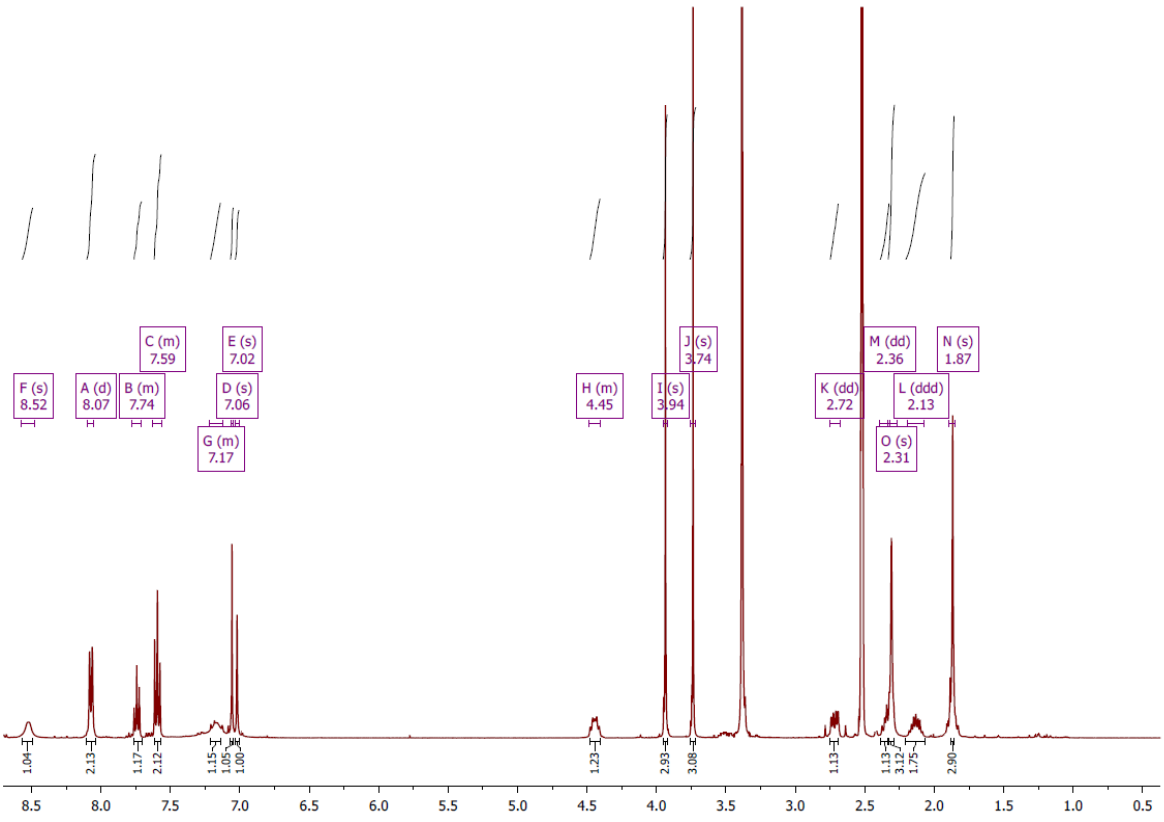

Figure S28. The ^{1}H NMR spectrum of **4d** in DMSO-d_6.

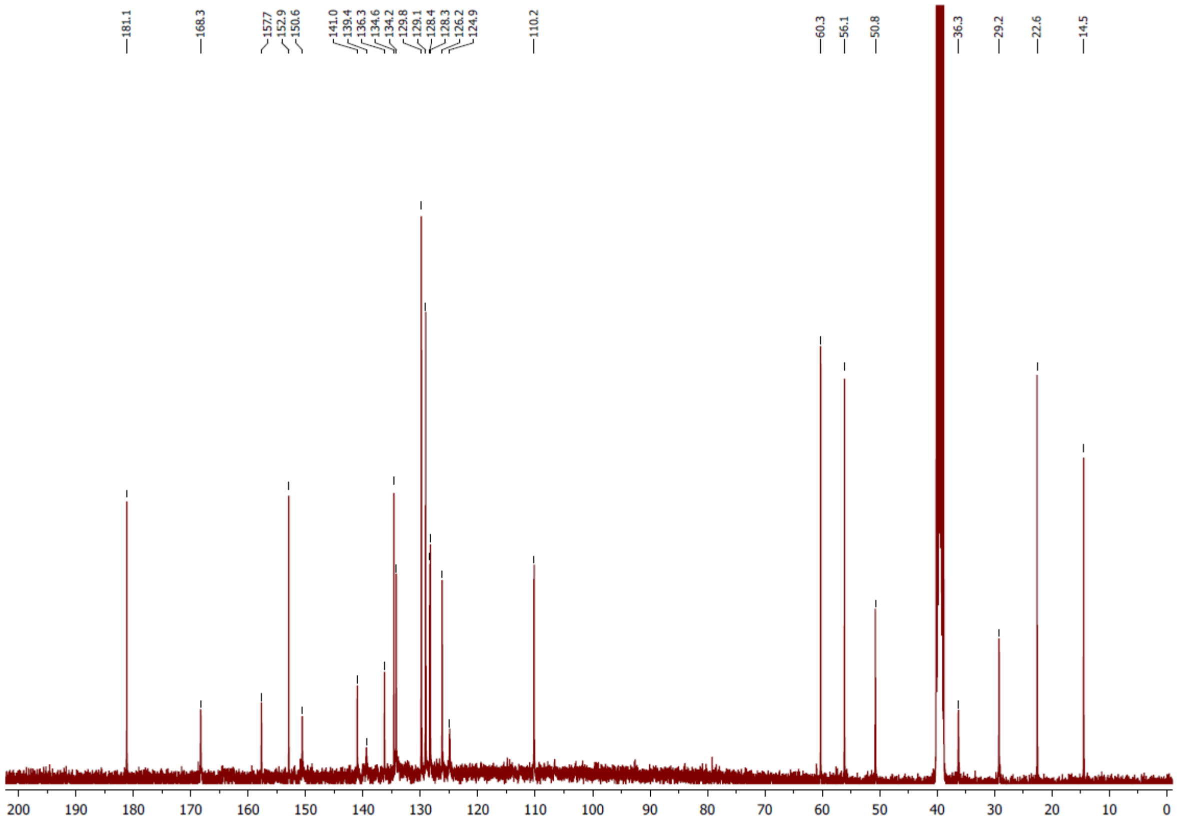

Figure S29. The ^{13}C NMR spectrum of **4d** in DMSO-d_6.

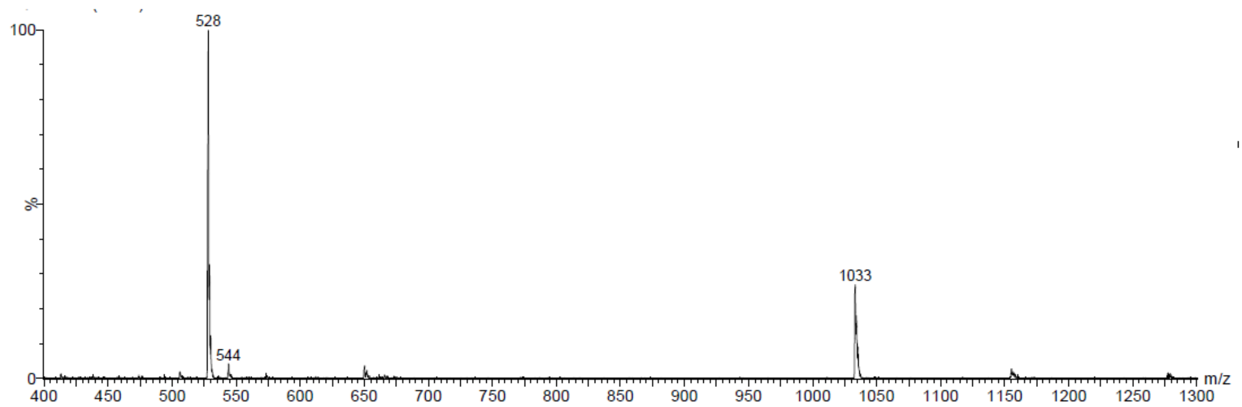

Figure S30. The ESI-MS spectrum of **4d**.

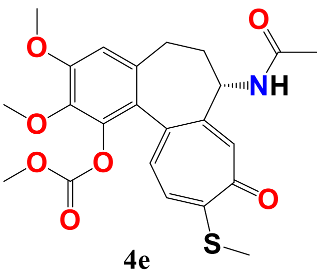

4e

Chemical formula: $C_{23}H_{25}NO_7S$, MW = 459,5 g/mol

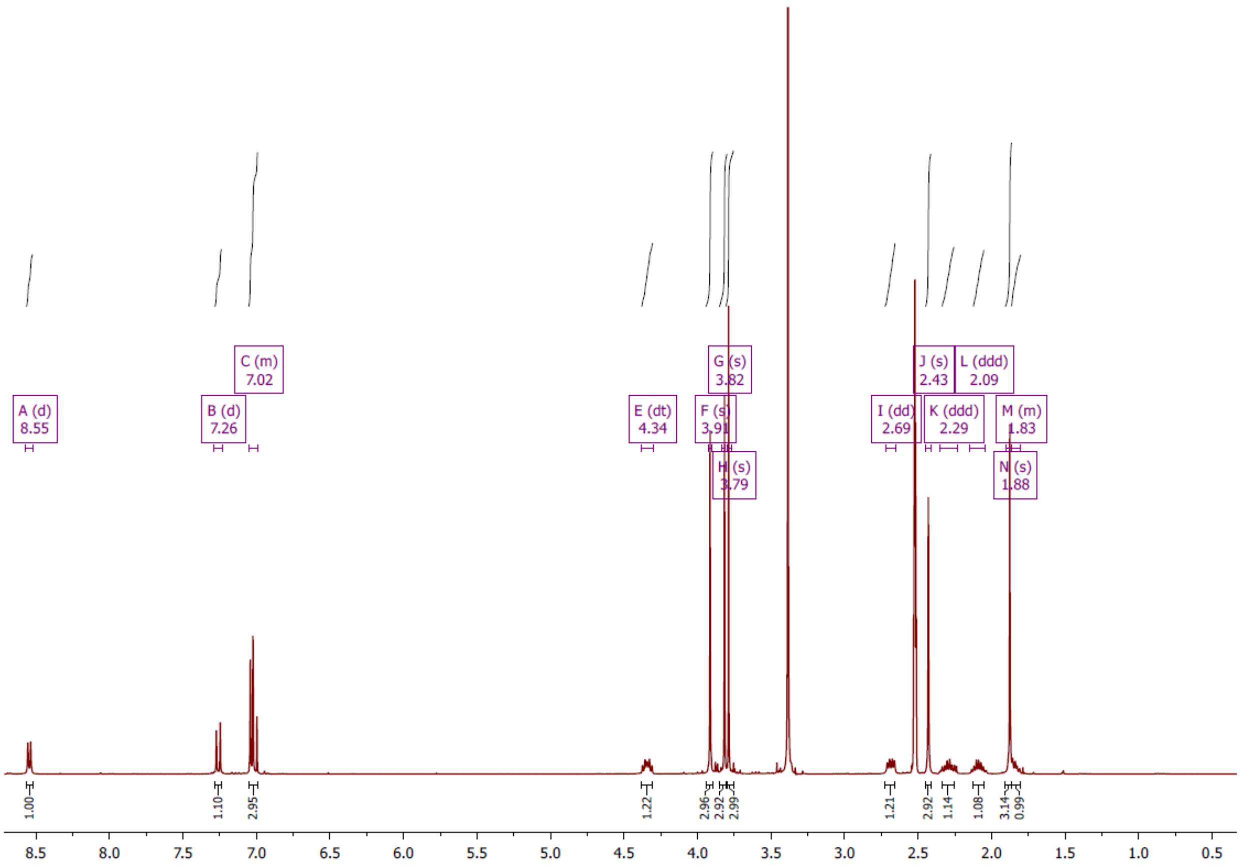

Figure S31. The ^{1}H NMR spectrum of **4e** in DMSO-d_6.

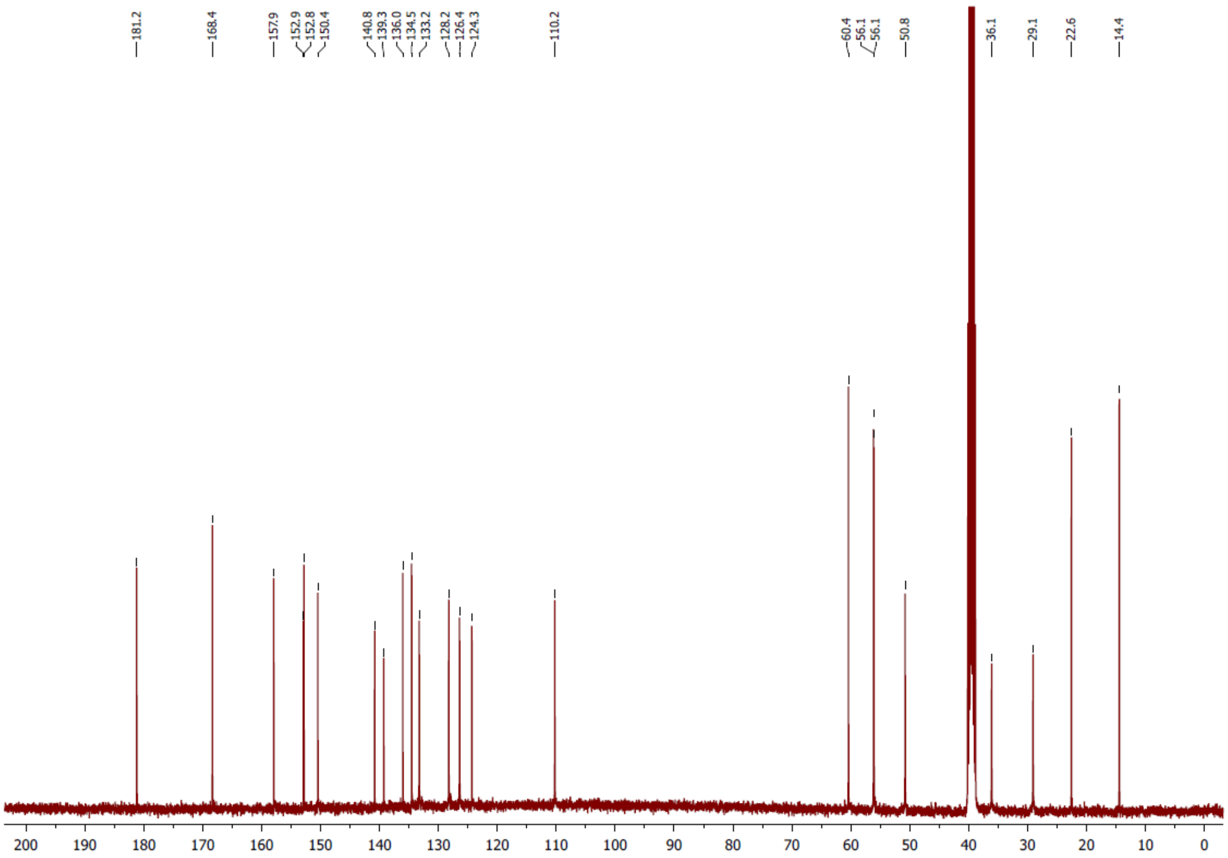

Figure S32. The ^{13}C NMR spectrum of **4e** in DMSO-d_6.

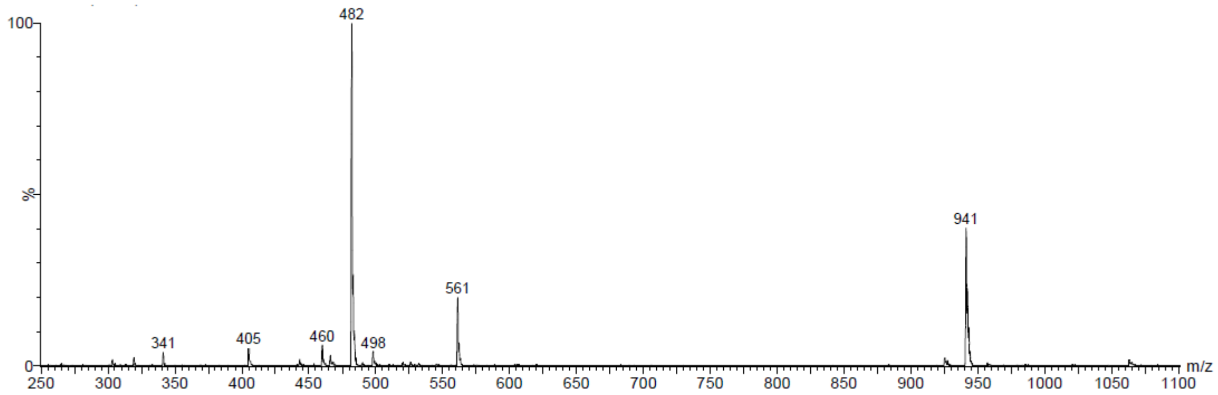

Figure S33. The ESI-MS spectrum of **4e**.

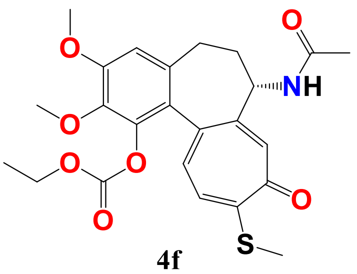

4f

Chemical formula: $C_{24}H_{27}NO_7S$, MW = 473,5 g/mol

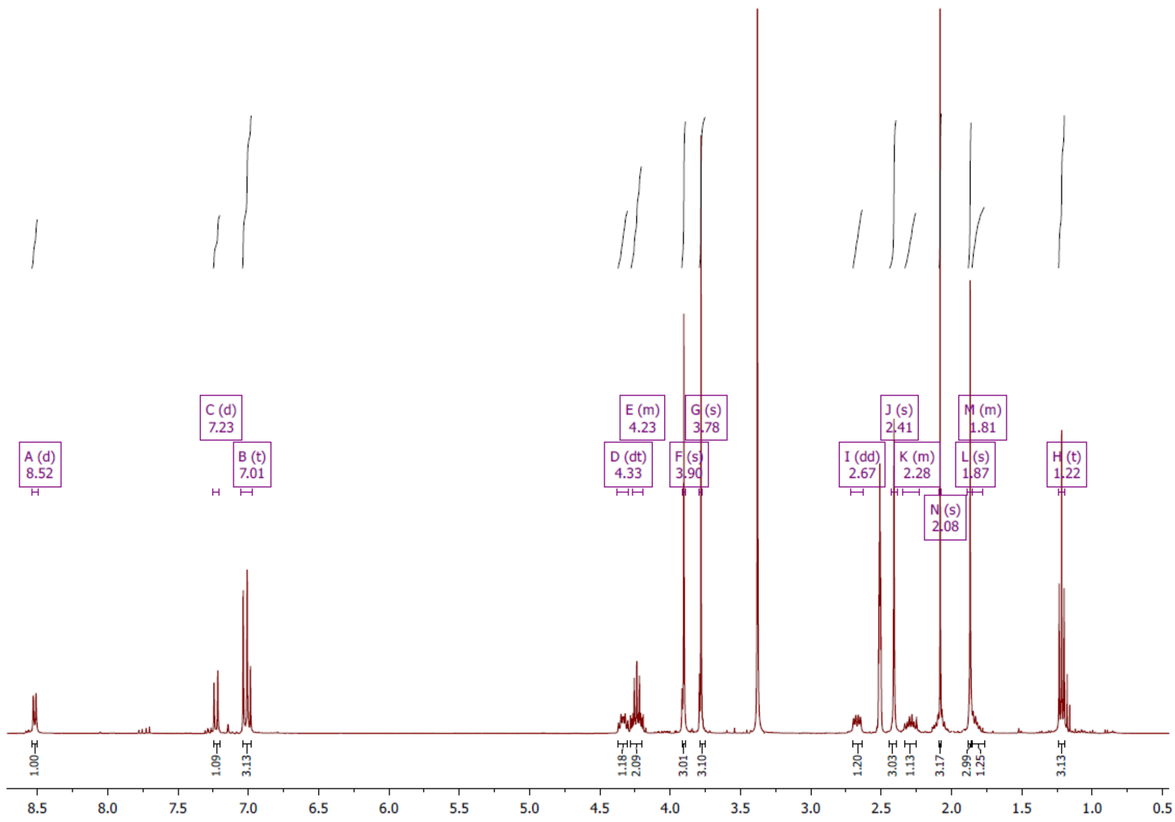

Figure S34. The ^{1}H NMR spectrum of **4f** in DMSO-d_6.

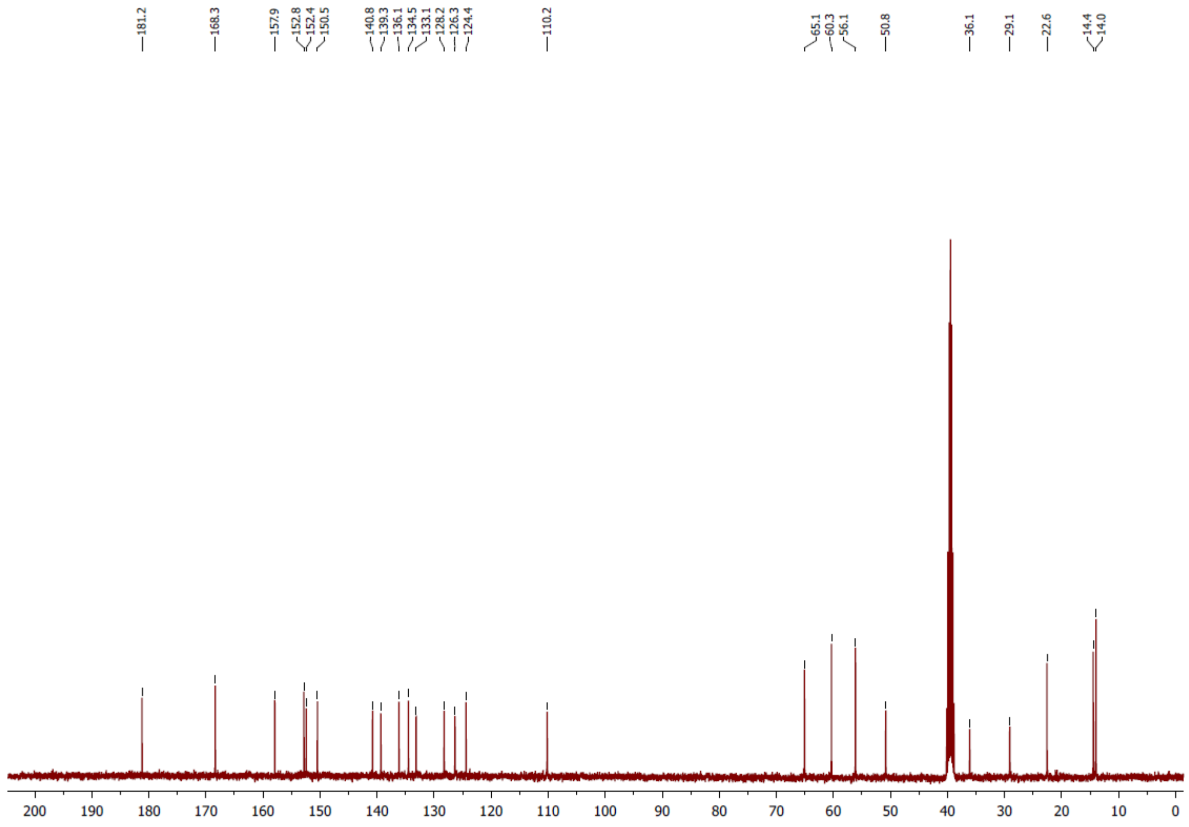

Figure S35. The ^{13}C NMR spectrum of **4f** in DMSO-d_6.

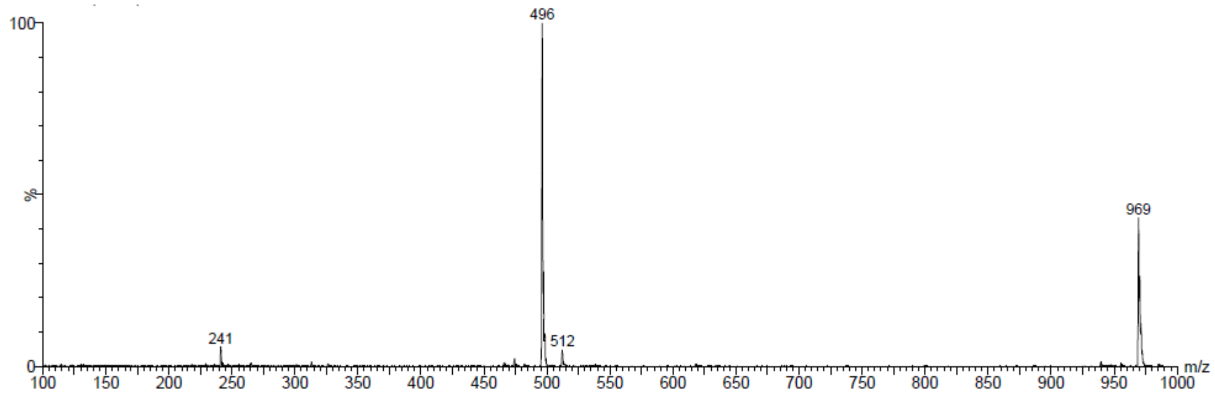

Figure S36. The ESI-MS spectrum of **4f**.

1. Skehan, P. *et al.* New colorimetric cytotoxicity assay for anticancer-drug screening. *J. Natl. Cancer Inst.* **82**, 1107–1112 (1990).

2. Nevozhay, D. Cheburator software for automatically calculating drug inhibitory concentrations from in vitroscreening assays. *PLoS One* **9**, (2014).

3. Xcalibur CCD System. CrysAlisPro Software System, version 1.171.33; Oxford Diffraction Ltd.: Wrocław, Poland. (2009).

4. Sheldrick, G. M. A short history of SHELX. *Acta Crystallographica Section A: Foundations of Crystallography* **64**, 112–122 (2008).

5. Sheldrick, G. M. Crystal structure refinement with SHELXL. *Acta Crystallogr. Sect. C Struct. Chem.* **71**, 3–8 (2015).

6. Dolomanov, O. V., Bourhis, L. J., Gildea, R. J., Howard, J. A. K. & Puschmann, H. *OLEX2* : a complete structure solution, refinement and analysis program. *J. Appl. Crystallogr.* **42**, 339–341 (2009).

7. Shi, Q., Verdier-Pinard, P., Brossi, A., Hamel, E. & Lee, K. H. Antitumor Agents-CLXXV. Anti-tubulin action of (+)-thiocolchicine prepared by partial synthesis. *Bioorganic Med. Chem.* **5**, 2277–2282 (1997).
